# Cutting edge advancements in biochar applications: bridging environmental remediation through adsorption and photocatalysis

**DOI:** 10.1039/d5ra06876b

**Published:** 2026-01-06

**Authors:** Amir Zada, Shohreh Azizi

**Affiliations:** a Department of Chemistry, Abdul Wali Khan University Mardan Khyber Pakhtunkhwa 23200 Pakistan amirzada@awkum.edu.pk; b UNESCO-UNISA Africa Chair in Nanosciences and Nanotechnology, College of Graduate Studies, University of South Africa Muckleneuk Ridge, PO Box 392 Pretoria 0002 South Africa azizis@unisa.ac.za

## Abstract

The contamination of the environment with toxins from both organic and inorganic resources has instigated numerous hazardous complications in plants and animals since the commencement of the industrial era. Many hazardous organics are very stable in both soil and water and can persist in the environment for a long time. The removal of these toxins is crucial because they are harmful. Biochar, as a low-cost material, is exceptionally useful in the purification and removal of hazardous materials. Herein, we discuss the role of biochar in the removal of hazardous materials from water. The adsorptive and photocatalytic removal of organic and inorganic contaminants, heavy metals, radioactive materials, antibiotics, phosphates and nitrates are discussed in detail. The mechanisms of adsorptive and photocatalytic removal of toxins are highlighted with suitable examples. The characteristics of biochar in light harvesting, charge utilization and adsorption of pollutants due to their porous nature, large surface area, and huge pore volumes are elaborated in detail. We believe that this article will attract substantial attention in the application of biochar for environmental remediation.

## Abstract 2

The contamination of the environment with toxins from both organic and inorganic resources has instigated numerous hazardous complications in plants and animals since the commencement of the industrial era.

## Graphical abstract

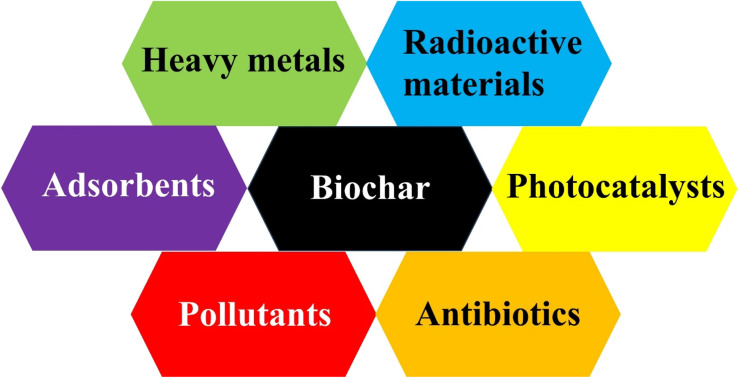

## 1. Introduction

Environmental pollution and energy crises are matters of extreme concern that have boomed substantially in the last few decades.^1–3^ Many organic compounds, including agricultural products, cosmetics, dyes, soap, detergents, effluents from hospitals, municipal committees and slaughterhouses, heavy metals from steel industries, electronic wastes, radioactive materials from nuclear plants and reactors, and explosive and related materials, have been added to the environment in large amounts in the past few decades.^4^ The race between different nations for technological and industrial developments has resulted in the addition of these complex and diverse industrial products to the environment. The magnitude of environmental pollution is very high in developing nations because of the scarcity of resources to address environmental contamination.

Currently, various pollutant eradication techniques, such as adsorption, coagulation, flocculation, filtration, membrane separation, osmosis, ozonation, electrocatalysis, and biological degradation, have been in practice for a long time and are used to remove unwanted substances from the aqueous environment.^5,6^ Advanced oxidation techniques, like Fenton reactions, are a new addition in this area to deal with the harmful and toxic pollutants present in the environment.^7^ Among these techniques, adsorption and photocatalysis are eye-catching techniques owing to their simple operational procedures, wide applicability, and high pollutant removal efficiency. Adsorption plays a significant role in regulating the distribution of hydrocarbons between the solid and liquid phases. It is found that adsorption generally controls the rate and extent of hydrocarbon migration between deep soil (solids) and groundwater.^8^ Adsorption does not even require light absorption or other sacrificial reagents and catalysts for the initiation of the removal process, and the process can be equally carried out even in the dark.^9^ However, selecting suitable adsorbents and photocatalysts is the most tedious task because most adsorbents are not universal and cannot be used to remove a wide range of pollutants.^10^

Biochar has several applications in the construction of buildings, energy generation and storage, soil treatment, carbon sequestration, wastewater treatment and environmental decontamination. It improves the mechanical strength and decreases the absorptivity of cement in the construction of buildings. It also plays an imperative role in the fertility of soil and the production of crops.^11^ Wastes, especially municipal sludge (though containing some vital nutrients such as C, P, N and K), retain a significant mass of hazardous materials, such as trace heavy elements, bacteria, viruses, polyfluoroalkyl organics, antibiotics, plastics, hormones, and other toxic compounds. By converting them into biochar, the hazardous performance of the waste can be significantly reduced.^12,13^ The reactivity of biochar exceptionally improves due to surface aging when it is coupled with organic wastes. Certain acidic and basic functional groups are formed due to the oxidation of biochar, which plays a crucial role in workable waste supervision to diminish the adverse effects of environmental waste organic matter. Coupling biochar with organic wastes reduces the availability of both C and N for greenhouse and acid rain-causing gases due to the high surface area and sorption performance of biochar.^14^ As a result, the interaction between biochar-organic wastes and soil improves, resulting in low soil density, improved water permeation, decreased loss of soil nutrients, and improved adsorption of pesticides and other necessary chemicals. The large surface area of biochar permits the retention of water and essential nutrients in the soil. It increases the pH of the soil to control soil acidity and provides essential nutrients for plants, reducing the discharge of both essential and non-essential substances from the soil.^15,16^

As a non-modified and modified material, biochar plays an exceptional role in wastewater treatment. It is a low-cost adsorbent and photocatalyst replacing expensive and unaffordable activated carbon-based adsorbents and photocatalysts for the removal of hazardous materials.^17,18^ The performance of biochar-based materials is evaluated based on the physiochemical characteristics of the raw feedstock and the methods of preparation.^19^ The commonly employed feedstock for the preparation of biochar-based adsorbents and photocatalysts includes plant and animal wastes, and municipal sewage sludge (from industry, forest, agriculture and municipality) owing to their natural abundance, environmentally friendly nature and physiochemical characteristics, such as large surface area, high porosity, excellent degree of unsaturation and high content of essential functional groups.^20,21^ The efficiency of biochar-based adsorbents and photocatalysts is determined from their surface characteristics, such as surface areas, pore volumes, availability of plentiful functional groups, and light absorption and charge separation. The presence of functional groups provides certain information about the mechanisms of pollutant removal and degradation, as the exclusion of toxins depends on the interaction and affinity of the biochar surface with the polarity and charge of pollutants. The formation of complexes between adsorbents and hazardous materials is largely influenced by the presence of functional groups.^22^ The presence of alcoholic, aldehydic and ketonic functional groups helps in the eradication of polar pollutants. Cations are easily attracted by negatively charged adsorbents, while anions are removed by positively charged adsorbents. The elemental composition of the adsorbents obtained from biochar also alters their performance. Since K, Mg, Ca, Al, Cl and Si are frequently present in the contents of biochar during carbonization of the raw materials, these elements may highly affect the adsorption efficiency, and corrosion of the equipment.^23,24^

In this article, we discuss the attributes of biochar for the preparation of suitable adsorbents and photocatalysts. The role of biochar in the adsorptive and photocatalytic removal and degradation of pollutants has been discussed in detail. Although several review articles are available highlighting the role of biochar in environmental purification, these articles either discuss the adsorptive removal of pollutants or highlight the photocatalytic removal of hazardous materials from the environment.^25,26^ Our article discusses the simultaneous removal of pollutants through both adsorption and photocatalysis. We hope that our article will highlight the application of biochar-based materials in environmental purification to prevent the accumulation of toxic materials on a large scale.

## 2. Treatment of raw biomass

Biomass is an organic matter that can be obtained directly or indirectly from plants and animals. By applying specialized techniques such as pyrolysis, hydrothermal, and gasification, biomass can be converted further into a solid, liquid and gaseous mixture. The solid portion is a useful matter called biochar, while the liquid and gaseous portions are termed crude and biogas, respectively. Biochar is an environmentally friendly porous solid carbon matter with outstanding applications as a fuel in energy generation and as an adsorbent in the removal of toxic substances.^27^ Owing to its limited and small surface area, poor porosity, and pore volume, untreated biochar is not effective in the removal of toxic materials from the environment. However, its surface area can be improved significantly by introducing certain activators through biological, physical and chemical methods to form a highly mesoporous and hierarchical structure for exceptional adsorption. Many inorganic acids, bases and salts are generally used to increase microspores, mesopores and macropores in biochar-based adsorbents for excellent adsorptive removal of toxic materials. The usefulness of biochar-based adsorbents mainly depends on their biomass source, production process, modification techniques and nature of activators.^28^ The following methods are generally anticipated to activate biochar-based materials for the preparation of excellent adsorbents to remove hazardous materials effectively.

### 2.1 Physical methods

Physical treatment is the most primitive procedure where biochar is heated at elevated temperatures between 700 and 900 °C for the development of porous structures in the presence of certain gases, such as steam, CO_2_, and air. In a strongly oxidative environment at high temperatures, auto-gasification of the material takes place, which significantly reduces the mass of volatile compounds and gases.^29,30^ As a result of the departure of volatile mass, pores of various natures (micropores, mesopores and macropores) develop in biochar under treatment, resulting in the strengthening of the surface area for excellent adsorption. However, the physical treatment of biochar is largely dependent on temperature; therefore, more caution should be devoted to monitoring and distributing temperatures for the required results. High temperature treatment also decreases the stability of activated biochar by rupturing the chemical bonds between the carbon atoms of the biochar. Further, the application of air generally promotes combustion of the carbon compounds of biochar, which drastically reduces the yield of biochar-based adsorbents.^31^ The formation of pores is not controlled by the physical treatment of biomass, as pores of different sizes and volumes are formed. However, the process is rapid and requires cheap equipment (heating assembly) and raw materials (water, air, CO_2_, *etc.*). The labor cost is low, and the results are excellent for the formation of high surface area adsorbents.^32^

### 2.2 Chemical methods

Chemical methods require the treatment of biochar under heating in the presence of certain chemical reagents to develop a porous material for excellent adsorption. Heating biochar between 400 and 950 °C in the presence of mineral acids (such as HNO_3_, H_2_SO_4_, H_3_PO_4_, and HCl), bases (such as KOH and NaOH), salts (such as CH_3_COOK, NH_4_Cl, ZnCl_2_, and KCN), and other reagents (like H_2_O_2_) generally induces two types of reactions: oxidation and dehydration.^33,34^ Both oxidation and dehydration processes result in the formation of micropores on the surface of biochar, which significantly improves the surface area and pore volume of the adsorbents. The efficiency of the formed adsorbents depends on the treatment temperature, nature and concentration of the chemical activator, and the types of biomasses. Although high risks are linked to the chemical treatment of the biochar, including a controlled mass of activator to prevent contamination due to excess mass, and corrosion of the equipment due to the presence of acids, bases and salts at high temperatures, the process is of great importance with significant control over the formation of pores.^35,36^ In most cases, low-temperature treatment provides excellent results. The yield of carbon-based materials is high because combustion reactions are highly restricted. Further, the stability of the formed adsorbents is very high, and they tolerate high-temperature treatment with excellent surface area and removal efficiencies.^37,38^

### 2.3 Biological methods

Biological methods involve the treatment of biochar with certain enzymes produced by bacteria, fungi, *etc.* The process is generally performed at room temperature, where lignin and cellulose from biochar are hydrolyzed to develop highly microporous materials suitable for the formation and use of effective adsorbents.^39,40^ The process is green and environmentally friendly and does not require foreign chemical activators for surface area enhancement. The carbon content is high, and these materials can also be used as fertilizers for the rapid growth of crops.^41,42^

## 3. Key physicochemical properties of biochar

Biochar is a highly porous material obtained from the pyrolysis of biomasses at elevated temperatures. The properties of biochar are vastly reformed with heating temperature, time and rate. The yield of the final product and their claims for the adsorptive deletion of pollutants are crucial and depend on the source from which the biomasses are selected for the formation of biochar.^43^ The following are some of the required properties that highlight the use of biochar as an adsorbent for the removal of toxic materials from the environment.

### 3.1 Pore volume and pore size of biochar

The adsorptive removal of pollutants depends highly on the availability of active sites on the surface of the adsorbents. The pore size and pore distribution play a decisive role in the adsorption of gases and pollutants to enhance the adsorption performance.^44^ Therefore, the presence of pores is crucial in biochar, as they act like a highway or buffer zone for the migration and transportation of particles to be removed from wastewater. Biochar is a highly porous material with a huge pore volume and pore size, demonstrating excellent adsorption and removal of toxic substances.^45^ The pyrolysis of biomasses at a temperature of more than 400 °C significantly improves the porosity of biochar due to the withdrawal of many volatile organics and gases. However, heating at a high temperature for a long time usually reduces the yield of the final product and therefore requires optimized heat conditions for a specific time.^46^

### 3.2 Polarity of biochar

Pollutants are either organic or inorganic materials. These pollutants carry either a positive or negative charge on their surfaces, or they may be neutral. Therefore, their interaction with the surface of the adsorbents is highly dependent on the various functionalities on the surface of the biochar. Polarity and aromaticity determine the ability and performance of adsorbents to adsorb pollutants of different natures. Biochar mainly contains elements like C, H, N, O, S, and P. The molar ratio between these elements can be used to determine whether a biochar-based adsorbent is polar or non-polar, and aromatic or non-aromatic.^47^ A lower H/C value validates that aromaticity is extraordinary due to complete carbonization, while a lower O/C value is responsible for lower polarity and excellent hydrophobicity to favorably remove non-polar and insoluble pollutants. Biochar obtained from biomasses of animal origin carries low carbon and high ash content with a significant amount of organic acids. Therefore, adsorbents obtained from such sources are polar in nature and help to remove polar and charged pollutants preferentially. The adsorbents obtained from plant-based biochar are highly porous with low polarity and can remove hazardous organic compounds.^48^ The presence of certain functional groups provides certain information about the mechanisms of pollutant adsorption. The formation of complexes between adsorbents and hazardous materials is largely influenced by the presence of functional groups. The presence of alcoholic, aldehydic and ketonic functional groups helps in the eradication of polar pollutants. Cations are easily attracted to negatively charged adsorbents, whereas anions are easily removed by positively charged adsorbents. Steam-based activation of biochar significantly advances oxygen functionalities to improve the polarity for the removal of charged pollutants.^49^

### 3.3 Specific surface area

Adsorbents with high surface areas are very efficient in eradicating large quantities of hazardous pollutants because they contain several active sites for anchoring particles. Biochar obtained from both plant and animal sources is highly porous, imparting large surface areas and active sites for the adsorption of pollutants. Their surface areas can further be boosted by activating biochar physically and chemically.^50^ Pyrolysis generally improves the surface area up to 500.0 m^2^ g^−1^ owing to the withdrawal of certain organic compounds and gases, such as CH_4_, CO_2_ and NO_*x*_. The departure of these compounds helps generate a porous structure with a large surface area and active sites for the removal of a large quantity of hazardous materials.

## 4. Applications of biochar

### 4.1 Biochar-based adsorbents

The gathering of toxic and hazardous ingredients in the soil, water and atmosphere has significantly disturbed the natural ecosystem, and the lives of many animals and plants are in severe danger. Many toxic chemicals and heavy metals from agricultural sites, industries, hospitals, and slaughterhouses are introduced into the environment every day. The removal of these substances is crucial for the survival of human beings, animals, and plants. In this section, we discuss in detail the removal of different toxic and hazardous materials from soil and water bodies with the aid of biochar-based adsorbents.

#### 4.1.1 Adsorptive removal of pollutants

**Fig. 1:**
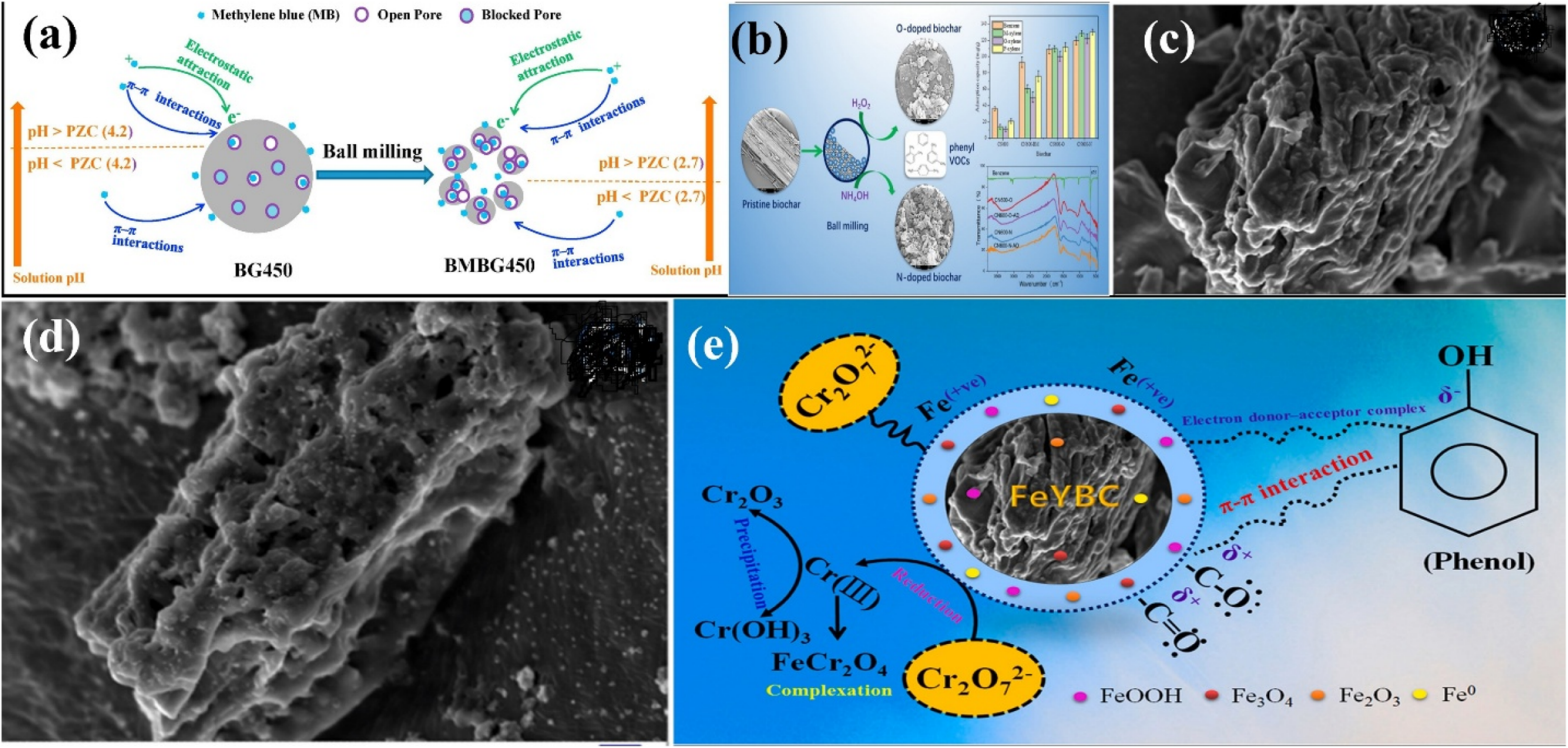
Mechanisms of methylene blue adsorption over un-milled and milled adsorbents (a). BG450 and MBBG450 represent un-milled and ball-milled biochar prepared from sugarcane bagasse biomass at 450 °C, respectively. Figure adapted/reproduced from ref. 53 with permission from Elsevier [(license number: 6145850910795) copyright 2025]; schematic of the preparation using ball milling technique (b). Figure adapted/reproduced from ref. 55 with permission from Elsevier [(license number: 6145860015651) copyright 2025]; SEM images of the pomelo peel-based pristine biochar (c) and iron oxide modified (d). Mechanism of Cr(vi) and phenol removal (e). YBC and FeYBC represent pristine biochar and iron oxide-modified biochar, respectively. Figure adapted/reproduced from ref. 56 with permission from Elsevier [(license number: 6145860266528) copyright 2025].

A variety of industries in developed and undeveloped countries across the world are responsible for their contribution to environmental pollution. Many organic pollutants are released into the external environment without proper checking and monitoring.^51,52^ These pollutants are very toxic, and their long presence in the environment causes fatal diseases in animals, especially aquatic animals. The removal of these toxic substances requires highly efficient low-cost adsorbents with large surface areas. Biochar-based adsorbents have been used extensively to adsorb organic pollutants from water and soil. The ball milling technique exceptionally improves the performance of the synthesized adsorbents. The physiochemical characteristics of ball-milled and un-milled biochar, such as the specific surface areas, pore volumes, and pH of the solution, greatly affect the removal of hazardous pollutants. Both the surface areas and pore volumes of the ball-milled adsorbents are superior to those of the un-milled biochar. Lyu *et al.* prepared adsorbents at different pyrolysis temperatures by selecting biomasses from various feedstocks (sugarcane, bamboo, and hickory chip wood) using the planetary ball milling technique for the removal of MB from wastewater to remove impurities.^53^ Interestingly, the ball milled process significantly improved the amount of oxygen (carboxyl, lactonic, and phenolic) functional groups on the surface of the adsorbents, which played an extremely central role in the removal of the pollutant. The adsorption of the pollutant increased from 216 to 318 mg g^−1^ when the pH of the solution was increased from 1.8 to 6.6 due to the increased strength of electrostatic and non-covalent π–π interactions between the adsorbent and pollutant. At the given pH value, adsorption was totally controlled by the π–π interaction between the adsorbent and pollutant, confirming that the graphitic structure slowly and gradually developed during ball milling progression. At a pH value of 7.5, both electrostatic and π–π interactions were operative to attain an adsorption of 392 mg g^−1^. Since the π–π interactions are weakened at higher pH values, it was concluded that electrostatic interactions mainly controlled the adsorption of methylene blue over the ball-milled biochar, as shown in Fig. 1a.

Pristine biochar-based adsorbents are boosted for improved adsorption by introducing certain functionalities, modifiers, and dopants. It has been found that the acidity of adsorbents improves with the introduction of oxygen functionalities. The O/C ratio increases significantly, confirming the high hydrophilicity of the adsorbents for the adsorption of polar pollutants.^54^ Zhang *et al.* used corn stalk to synthesize H_2_O_2_ and NH_4_OH modified efficient adsorbents at 600 °C for the adsorption of phenyl-based volatile organic pollutants, as shown in Fig. 1b.^55^ Specific surface area measurement indicated that the pore size of the adsorbents was reduced from 4.68 to 1.94 nm, and the surface area was significantly improved from 168.24 to 405.61 m^2^ g^−1^ during the ball milling process, suggesting that this technique not only enlarges the exterior surface area by slashing it into very small masses but also improves the micropores and mesopores by opening the previously closed porous network as provided in the SEM images. The increased O/C ratio from 0.14 to 0.36 confirmed the high hydrophilicity of the prepared adsorbents to adsorb polar pollutants. The improved adsorption is related to the modification of biochar with NH_4_OH, which replaces oxygen functionalities with amino functionalities on the surface of the adsorbents.

Iron-modified pomelo peel-based adsorbents were fabricated by Dong *et al.* to eliminate phenol and dichromate anions from aqueous solution.^56^ The SEM images confirmed the porous morphology of the pristine biochar and small iron oxide particles on the surface of the iron oxide-modified adsorbent, as shown in Fig. 1c and d. The concentrations of carbon and oxygen changed from 78.63 and 20.44% to 58.16 and 22.29%, respectively, after the arrival of the iron oxide modifier. The O/C ratio of the modified adsorbent was higher than that of the pristine adsorbent, confirming that the modified adsorbent was able to adsorb oxophile substances significantly. The positive zeta potential of iron oxide-modified biochar was responsible for attracting dichromate anions significantly. The adsorption of phenol and dichromate anions was also investigated in the presence of NaCl. Interestingly, the adsorption of phenol was not greatly affected by the presence of NaCl. However, the adsorption of dichromate anions was greatly influenced by the concentration of NaCl. As the amount of NaCl increased, the adsorption of the dichromate anions greatly decreased due to the hindrance effect caused by the Na^+^ ions. The whole mechanism is illustrated in Fig. 1f.

**Fig. 2:**
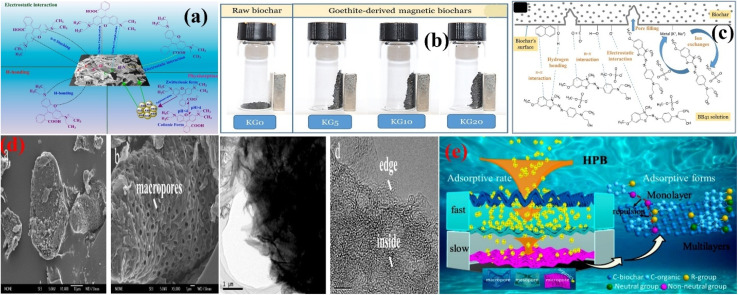
Schematic of the adsorption mechanism of RhB over the Ag-loaded biochar-based adsorbent (a), figure adapted/reproduced from ref. 57 with permission from Elsevier [(license number: 6145860428334) copyright 2025]. Digital photograph of the adsorbents in a magnetic field (b), proposed adsorption mechanism of basic blue 41 (BB41) over the kelp-goethite biochar (KG_5_) (c). (KG_0_, KG_5_, KG_10_ and KG_20_ respectively) Show pristine kelp-goethite biochar and biochar treated with 5%, 10%, and 20% goethite mineral, and the mechanism of adsorption of BB41 on KG_5_ (c), figure adapted/reproduced from ref. 59 with permission from Elsevier [(license number: 6145860618577) copyright 2025].^59^ FE-SEM images [(a and b)] and HR-TEM images [(c and d)] of the HPBC (d). Schematic of the adsorption of pollutant (e). Figure adapted/reproduced from ref. 60 with permission from Elsevier [(license number: 6145860831879) copyright 2025].

Biochar-based adsorbents obtained from algal-based biomass are enormously effective because they carry certain groups that are very effective in adsorption. Shaikh *et al.* obtained Ag-modified adsorbents from tropical freshwater Spirogyra macroalgae at 350 °C for 3 h using the ball milling method for the eradication of rhodamine B from wastewater by varying adsorbent dosage, pollutant concentration, temperature, pH, and contact time.^57^ The negative value of Δ*G*° suggested that adsorption was spontaneous and exothermic. The effect of pH was more pronounced, and a maximum amount of 46.38 mg g^−1^ was removed at a pH value of 10. The FTIR study of the adsorbents before adsorption confirmed the presence of sulfoxide (S=O), saturated and unsaturated aldehydes and ketones, alkynes, amine, and phenol functionalities, along with peaks related to Ag nanoparticles. Interestingly, after the adsorption of dye, the FTIR peaks related to sulfoxide and aldehydes and ketones were significantly reduced in their intensities with obvious shifting probably because of the π–π communication between the adsorbents and adsorbate. Further, the FTIR peak related to the amine functional group was shifted to confirm the hydrogen bonding mechanism behind rhodamine B adsorption. Pseudo-first order (SFO), pseudo-second order (SSO) and intra-particle diffusion (IPD) models were applied to monitor the kinetics and mechanisms.^58^ The kinetic models suggested chemisorption of the rhodamine B dye over the prepared adsorbents, involving IPD and electrostatic interaction, as shown in Fig. 2a.

To improve the separation of biochar-based adsorbents, magnetic materials have been modified by many researchers. Sewu *et al.* used one of the thermodynamically stable and abundant oxides of iron called goethite (α-FeOOH) to modify biochar-based adsorbents for the efficient adsorption of pollutants and separation.^59^ Saccharina japonica was impregnated with goethite mineral under stirring for 24 h and pyrolyzed at 600 °C for 1 h. The prepared adsorbents were employed to remove basic blue 41 from wastewater, as shown in Fig. 2b. The physicochemical properties of the adsorbents suggested that the addition of goethite had almost no effect on the pH of the biochar because the net surface charge was constant under all loading of goethite. However, the amount of elements, such as K, Na, Ca, and Mg (responsible for cation exchange with dyes), was reduced except for Al, while those of elements, like Si, Fe, S, and P, were improved after the introduction of goethite. The dye elimination efficacy of the goethite provoked adsorbents was significantly better than that of the pristine ones. Again, a high potential was established on the surface of the adsorbent for the repulsion of the dye at a low pH. As the pH increased, more OH^−^ ions were liberated in the solution to convert the positive surface potential into negative for the electrostatic attraction of the cationic dye. The application of H-bonding was confirmed from the FTIR study with a significant peak shifting of the OH group to a higher wavenumber. A shift in the stretching vibration of the aromatic ring to a lower wavenumber suggests the services of π–π interaction. The disappearance of the FTIR peak related to C=O indicated that n–π interaction was also operative in the adsorption of the dye. Further, the low desorption efficiency of the dye in NaCl solution and the unrecovered adsorption capability of the adsorbents after treatment with HCl and KCl solutions confirmed the involvement of electrostatic interactions and ion exchange mechanism, respectively, as shown in Fig. 2c.

Although many authors have contributed significant information towards the adsorptive behavior of biochar-based adsorbents by simply improving their porosities, the reported literature is limited to the regulation of pore structures. It has been found that increased porosity does not always ensure the enhanced adsorption capability of the adsorbents. In many cases, a slow adsorption speed ranging from several hours to many days is the limiting factor for the large-scale application of the reported adsorbents. It is believed that the preparation of biochar with multi-level pores is more important than improving the concentration and volume of the pores. Yu *et al.* used shrimp shell biomass for the preparation of hierarchical porous biochar (HPBC) using thermal cracking and acid treatment techniques to remove 2,4-dichlorophenol, rhodamine B, and tetracycline hydrochloride from wastewater at a super-fast adsorption speed.^60^ The biomass was heated at 800 °C under N_2_ atmosphere. The as-obtained pristine adsorbent was treated with HCl to form the HPBC. Macropores of more than 50 µm were detected in the SEM/HRTEM images with smooth surface morphology of HPBC inclined to be crumpled like graphene, as shown in Fig. 2d. Impurities in the starting biomass (Na, Mg, Ca, P and Cl) acted as a pore template that condensed and grew into equally dispersed large-sized micropores and mesopores. From the adsorption study, it was confirmed that no matter the concentration and type of the pollutants, the super-fast adsorption equilibrium was reached in 10 min. An SSO kinetic model confirmed the chemisorption of the three pollutants. The adsorption of the pollutants comprised two parts. In the first part, the particles of pollutant migrated from the solution to the surface of the adsorbent, and this process was monitored by molecular and film diffusion. In the second part, further diffusion of the pollutant took place in the pores of the adsorbent. From the Langmuir and Freundlich models, the adsorption of rhodamine B and tetracycline hydrochloride was monolayered, while that of 2,4-dichlorophenol was multilayered. Based on the structure and nature of the pollutants, three main contributors in the forms of charged sites (electrostatic interaction), benzene ring (π–π interaction) and N- or O-functionalities were considered to investigate the adsorption mechanism, as shown in Fig. 2e.

#### 4.1.2 Adsorptive removal of antibiotics

In the past few decades, significant control has been achieved over many fatal diseases by discovering broad-spectrum antibiotics and vaccines.^61^ However, the disposal of these antibiotics into the environment from pharmaceutical industries, hospitals, and farmlands has created huge environmental and medical issues.^62^ The presence of these antibiotics in water generates many drug-resistant bacteria and pathogens; therefore, infectious diseases have become more fatal with a rise in total deaths in the past few decades.^63^ In the USA, about 2.8 million antibiotic-resistant infections occur every year, with a total death rate of more than 35 000. However, in the European Union, China, India, Pakistan, the Middle East and Africa, the situation is more adverse. Among the available antibiotics, bacterial infections caused by penicillin and cephalosporin-based resistive bacteria are responsible for more than 55% of infections, with more than 40% attributable deaths.^64^ The world has recently seen not only the deadly impacts of COVID-19 but also the dormancy of many economies throughout the world. Thus, it is a significant issue to deal with these pollutants in the earliest stage to prevent the mass generation of antibiotic-resistive bacterial genes and severe environmental and medical issues.

**Fig. 3:**
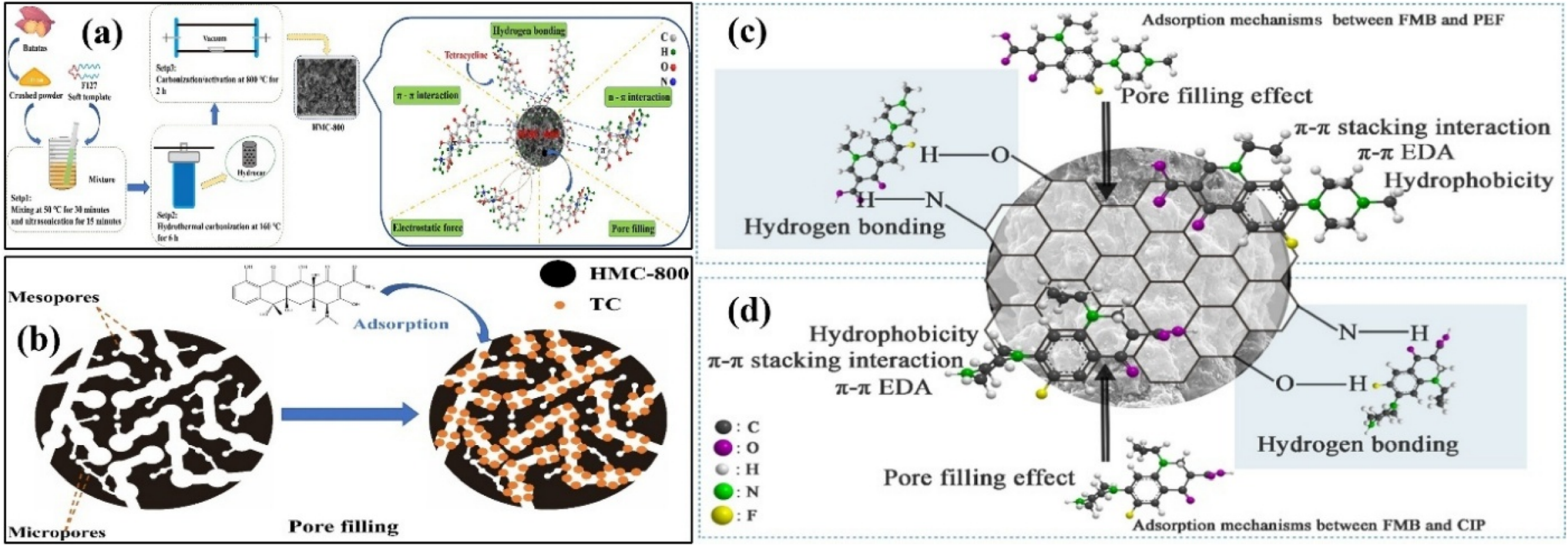
Preparation of adsorbent from the batatas-based biomass (a) and schematic of the pore filling mechanism of TC adsorption (b). HMC-800 denotes the template-based adsorbent prepared at 800 °C. Figure adapted/reproduced from ref. 66 with permission from Elsevier [(license number: 6145861051550) copyright 2025]. Schematic of the adsorption mechanisms of PEF (c) and CIP adsorption onto FMB (d). Figure adapted/reproduced from ref. 67 with permission from Elsevier [(license number: 6145861192056) copyright 2025].

Biochar-based adsorbents have gained much interest in the removal of antibiotics owing to their ease of formation, availability of waste biomass for their preparation, highly porous morphology, and long life span. The interaction of antibiotics and, hence, their adsorptive removal depends on the hydrophobicity of the working adsorbents.^65^ Zheng *et al.* prepared a highly efficient adsorbent from batatas-based biomass for the removal of tetracycline antibiotic (TCA).^66^ A pristine adsorbent was prepared without the assistance of the soft template, as shown in Fig. 3a. The removal of TCA was monitored under different pH values. The amphoteric TCA was found in four forms (H_4_T^+^, H_3_T, H_2_T^−^ and HT^2−^) in aqueous solution. Additionally, the pH_PZC_ value of the template-based was 3.69; therefore, the surface of the adsorbent was positive when pH < 3.69 and was negative when it was greater than 3.69. When the pH was between 3.0 and 11.0, the adsorption of H_2_T^−^ and HT^2−^ was mainly hindered by electrostatic repulsion between the TCA and adsorbent. When the pH increased between 5.0 and 7.0, the removal of TCA was gradually enhanced, as it is normally found to have a zwitterion with a weak charge. For the kinetic study, SFO, SSO and IPD models were studied. The application of the SSO model realized a sizable non-linear *R*^2^, and the theoretical adsorption was close to the experimental adsorption compared to the SFO model. The FTIR and Raman studies provided significant clues about the H-bonding and n–π mechanisms. A significant shift in the FTIR peak of –OH authorized the formation of H-bonding between the adsorbent and adsorbate. Additionally, a shift in the C–O peaks to a larger wavelength justified the n–π interaction as an important contributor to TCA adsorption, as shown in Fig. 3b.

Fluoroquinolone belongs to a class of antibiotics mainly used against respiratory infection causing bacteria. For the adsorptive removal of ciprofloxacin and pefloxacin (members of fluoroquinolone) from wastewater, Xiang *et al.* fabricated Fe- and Mn-loaded adsorbents from vinasse (waste from sugar cane industry).^67^ The physiochemical characterization confirmed that the as-prepared adsorbent possessed a pore size, pore volume and surface area of 1.630 nm, 0.127 cm^3^ g^−1^ and 94.943 m^2^ g^−1^, respectively, with irregular morphology and excellently developed porosities showing well-distributed MnFe_2_O_4_ and Fe_3_O_4_ particles on the surface. When the pH was increased, a significant increase in adsorption efficiency was noted. Adsorption of both antibiotics was realized by fitting the data into the SFO, SSO, Elovich, and Bangham models. Complete control of the heterogeneous diffusion factor was densely linked with the Elovich model. From the adsorption isotherms, it was found that the experimental data were satisfactorily explained by the Freundlich isotherm for both antibiotics. The hydrophobicity of both antibiotics was studied to determine their interaction with the surface of the adsorbent. Obviously, the hydrophobicity of pefloxacin was higher than that of ciprofloxacin, indicating that the former was more frequently adsorbed on the surface of the Fe/Mn modified biochar-based adsorbent. The adsorption mechanisms of both antibiotics are shown in Fig. 3c and d.

**Fig. 4:**
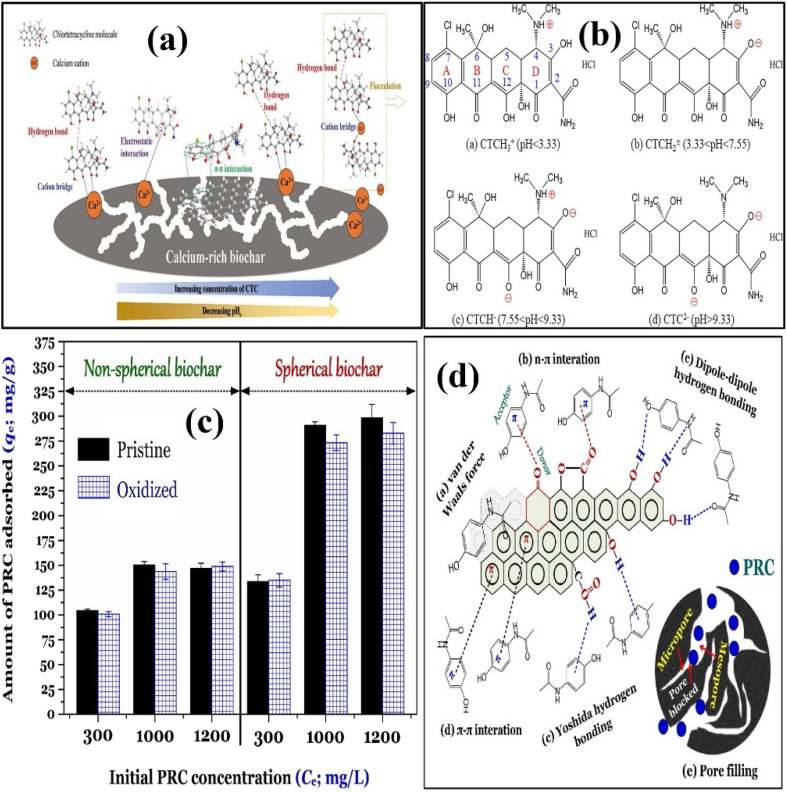
Preparation of the Ca-rich adsorbent from crab shell and mechanism of Cl-ciprofloxacin adsorption (a); net charge on Cl-ciprofloxacin at different pH values (b). Figure adapted/reproduced from ref. 68 with permission from Elsevier [(license number: 6145870065307) copyright 2025]. Evaluation of adsorption of PRC (c); adsorption mechanism of paracetamol over SB and NSB (d). Figure adapted/reproduced from ref. 69 with permission from Elsevier [(license number: 6145870267358) copyright 2025].

Biochar-based adsorbents are not only produced from plant residues, but animal residues are also very effective in the generation of highly porous adsorbents for the real eradication of objectionable substances from wastewater. Basically, it has been accepted that adsorption is mainly completed in four stages: (i) diffusion of the adsorbate particles into the liquid phase, (ii) migration of particles from liquid phase to the surface of adsorbent, (iii) diffusion of the adsorbate particles into the internal pores of the adsorbent and (iv) occupation of the adsorbate particles on the external and internal active sites of the adsorbent. Crab shell-based crustaceous biomass was used by Xu *et al.* to prepare a Ca-rich adsorbent for the removal of Cl-ciprofloxacin antibiotics, as shown in Fig. 4a.^68^ The percentage amounts of C, N, H and Ca were found to be 11.29%, 1.62%, 1.62% and 36.14%, respectively. An eradication efficiency of 90% was attained in just 20 minutes, while adsorption equilibrium was accomplished in 120 minutes, indicating the potential of the prepared adsorbent for environmental purification. SFO, SSO and IPD models were explored for the fitting of kinetic data. In this study, the authors confirmed that the SSO model had a better fit than the SFO and IPD models, demonstrating that adsorption was dominated by chemosorption. The inorganic carbonaceous part of the crab shell results in the formation of basic CaO during the pyrolysis of the biomass, thereby increasing the pH of the suspension to 12.36. The pH_pzc_ calculated for the prepared Ca-rich crab-shell-based adsorbent was 6.70, indicating that a pH value lower than 6.70 establishes a positive charge, while a pH value higher than 6.70 introduces a negative charge on the surface of the adsorbent. Thus, at lower pH values, the adsorption of the antibiotic was lower due to the mutual repulsion between the adsorbate and adsorbent. As the pH increased, more Cl-ciprofloxacin particles accumulated on the adsorbent surface to boost the removal efficiency, as shown in Fig. 4b. Tran *et al.* prepared spherical and non-spherical biochar-based adsorbents for the abolition of paracetamol from wastewater.^69^ Spherical biochar (SBs) was prepared from glucose following a two-step synthetic procedure, while non-spherical biochar (NSBs) (counterparts of the spherical adsorbents) was prepared by directly heating the pomelo peel-based biomass in a one step process. The FTIR study confirmed the presence of hydroxyl groups due to phenolic and carboxylic acid functional groups, aromatic, lactonic, and ether on the surface of both types of adsorbents. The amount of paracetamol adsorbed continuously increased as the contact time increased. About 62% and 56% paracetamol were removed with SB and NSB in 2 min, respectively. These enhanced efficiencies demonstrated that both types of adsorbents possessed excellent attraction to the target pollutant. SFO, SSO and Elovich models were employed to monitor the adsorption kinetics. Several factors (such as H-bonding, electrostatic and van der Waals interactions, pore filling, n–π and π–π interactions) have generally contributed to the adsorption of aromatic compounds with some polarity on the surface of the adsorbents. The magnitude of electrostatic and van der Waals interactions was relatively low because paracetamol was not ionized at a neutral pH. The role of π–π interaction was followed by oxidizing both types of adsorbents with HNO_3_, as shown in Fig. 4c. Since the oxygen containing functional groups act as electron withdrawing groups, a significant reduction in the charge density in aromatic rings reduces the intensity of π–π interactions. However, the adsorption efficiencies of both types of adsorbents changed slightly after oxidation, suggesting the integral role of π–π interactions in the adsorption of paracetamol over the prepared adsorbents, as shown in Fig. 4d.

#### 4.1.3 Adsorptive removal of heavy metals

**Fig. 5:**
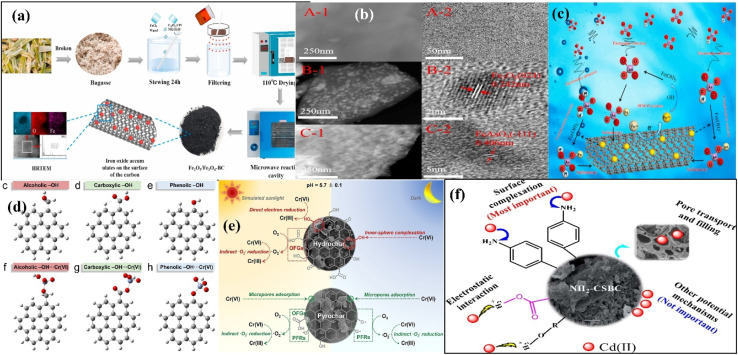
Preparation of iron oxide modified biochar (a); TEM and HRTEM images of the pristine adsorbent before adsorption [(A1–A2)], iron oxide modified biochar [(B1–B2)] and iron oxide modified biochar after adsorption [(C1–C2)] (b); and schematic of the As adsorption (c). Figure adapted/reproduced from ref. 71 with permission from Elsevier [(license number: 6145870543559) copyright 2025]. Atomic clusters of hydrochar with the alcoholic [(c)], carboxylic [(d)] and phenolic hydroxyl groups [(e)] (d). Inner-sphere complexes of Cr(vi) with alcoholic [(f)], carboxylic [(g)] and phenolic hydroxyl groups [(h)] with hydrochar. Gray, white, red, and purple spheres represent C, H, O and Cr atoms, respectively. Structurally dependent Cr(vi) removal processes (e). Figure adapted/reproduced from ref. 73 with permission from Elsevier [(license number: 6145870776927) copyright 2025]. Schematic for the synthesis of modified biochar (NH_2_-CSBC) (a), and adsorption of Cd(ii) over it (f). Figure adapted/reproduced from ref. 74 with permission from Elsevier [(license number: 6145870926521) copyright 2025].

Many metallurgical, chemical, and agricultural industries are discharging their wastes directly into natural water bodies. The effluents of these industries contain huge quantities of heavy metals, such as Cr, Cu, As, Cd, Pb, and Ni, causing huge environmental concerns that jeopardize animal health and plant life.^70^ Many of these metals are carcinogenic and are responsible for cancer diseases in the skin, lungs, and urinary tract. Many other diseases found in adult patients, including hypertension, and cardiovascular and respiratory tract diseases, are directly influenced by different heavy metals in drinking water bodies. Therefore, it is necessary to deal with these metals by establishing efficient, environmentally friendly technologies. Currently, the elimination of metals from water majorly depends on various techniques, such as the ion-exchange method, chemical precipitation method, and membrane separation technique. However, these methods have many shortcomings, including the generation of secondary pollutants, interference of the ions with the ion-exchange column and high cost of the materials in the membrane separation technique. In contrast, the adsorptive removal of heavy metals is fast, low-cost, easily recyclable and environmentally friendly. However, the accomplishment of adsorption depends on synthesizing competent adsorbents at a low cost from renewable precursors to eradicate large quantities of heavy metals. Agricultural wastes are of great significance in this regard for providing highly porous and efficient adsorbents because they are nontoxic and biodegradable and contain large quantities of cellulose, lignin, and hemicellulose to fabricate biochar-based adsorbents and eliminate heavy metals. However, the major issue with these adsorbents is their separation from the liquid–solid mixture after the completion of the adsorption process owing to their fine particle size. Many authors have tried to improve separation efficiency by introducing certain additives, such as iron oxide, owing to its excellent magnetic behavior. The abstraction of heavy metals from wastewater through adsorption largely depends on the nature of the metal and the intrinsic properties of the adsorbents. Therefore, investigation of the structural-dependent adsorption activity of adsorbents for the elimination of heavy metals is crucial to realize maximum pollutant eradication efficiency in a short time. Bai *et al.* used sugarcane waste as a raw material for the formulation of iron-modified adsorbents to eradicate As(v) from aqueous solution, as shown in Fig. 5a.^71^ The SEM and TEM images of the prepared adsorbent are shown in Fig. 5b. Thermodynamic parameters, such as adsorption enthalpy and entropy, were premeditated from Langmuir and Freundlich isotherms. Interestingly, the removal of As was greatly influenced by many factors, such as surface area, porosity, pore size distribution, surface functionalities of the adsorbent and loading mass of iron oxide. As the dosage of adsorbent was enhanced from 0.1 to 1.0 g, the metal eradication capability increased from 27% and 93% to 64% and 96%, respectively. Interestingly, the adsorption of the metal was negligible when the pH value was less than 5. The effect of temperature was also investigated, and it was found that a change in temperature (25–45 °C) has almost no effect on the adsorption rate of the metal. Based on the values of *R*^2^, it was found that chemical adsorption was the dominant process when the initial concentration of As was increased. It has been found that adsorption of As over the prepared adsorbent was energized by both physical and chemical adsorptions with the aid of three interactions: van der Waals interaction, redox reaction, and co-precipitation. As is present in the solution as arsenate (H_3_AsO_4_, H_2_AsO_4_^−^, HAsO_4_^2−^, and AsO_4_^3−^). During redox reactions, Fe^2+^ is oxidized to Fe^3+^, followed by the precipitation of Fe(OH)_3_. The formed Fe(OH)_3_ reacts with arsenates to form different iron arsenates adsorbed on the surface of the adsorbent. In co-precipitation, arsenates react with ferrite to form a precipitate of iron arsenates and are deposited on the adsorbent to realize chemical adsorption. The whole mechanism is shown in Fig. 5c. Chen *et al.* prepared two types of adsorbents (pyrochar and hydrochar) through pyrolysis and hydrothermal carbonization for the removal of Cr(vi).^72^ Pyrochar was enriched in carbon content due to its high carbonization temperature (350–700 °C), while hydrochar contained a high degree of oxygen functionalities realized through the hydrolysis of the precursor materials in water.^73^ When the Cr(vi) solution was mixed with hydrochar in the dark, only a 19% reduction in its concentration was detected in 8 h. However, when the same experiment was conducted using a pyrochar-based adsorbent, the concentration of Cr(vi) was significantly reduced with a corresponding increase in the concentration of Cr(iii) in the dark. This suggested that the pyrochar-based adsorbent was suitable to follow the reduction pathway for the removal of metal in the dark although some amounts of Cr(vi) were also removed through adsorption. However, both the adsorbents show significant metal removal efficiencies in sunlight as the concentration of Cr(vi) was decreased significantly, while that of Cr(iii) was increased in the case of both hydrochar and pyrochar-based adsorbents. Interestingly, the removal efficiencies and rates of reduction were significantly reduced after the eradication of O_2_ in the case of both adsorbents in the dark and in sunlight, suggesting that O_2_ was favorably adsorbed on the surfaces of both adsorbents to receive electrons and generate super oxide anions for the reduction of the metal. Since electrostatic interaction and diffusional migration control physical adsorption, both mechanisms were investigated in detail at an experimental pH of 5.70. Under these conditions, Cr is normally present in the form of Cr_2_O_7_^2−^, CrO_4_^2−^ and HCrO_4_^1−^. During adsorption, the occupation of Cr may substitute for –OH groups to create an inner–sphere complex. Since there are three –OH containing functional groups (alcoholic, carboxylic and phenolic) on the surface of the prepared adsorbents, it was necessary to find which –OH was involved in the formation of complex and was confirmed from the DFT investigations, as shown in Fig. 5d. It was confirmed that the Mulliken charges of the aromatic rings containing alcoholic and carboxylic –OH were the same before and after complexation, while those containing phenolic –OH were enhanced after inner sphere complex formation, as shown in Fig. 5e. This confirmed that charge density was reduced on the ring carrying phenolic–OH; therefore, the adsorption of Cr over the hydrochar-based adsorbent was chemical in nature through inner–sphere complex formation between the metal and adsorbent. The introduction of organic functional groups in biochar-based adsorbents is extremely beneficial for improving their adsorptivity for many heavy metals. Ma *et al.* used corn stalk biomass for the preparation of biochar.^74^ For nitrification, the pristine biochar was treated with a mixture of H_2_SO_4_ and HNO_3_ under stirring, followed by refluxing with NH_4_OH and Na_2_S_2_O_4_ solution to obtain a modified biochar-based adsorbent for the removal of Cd. A comparison of the properties of pristine and modified biochar indicated that the concentrations of C and H were reduced, but that of N was increased after the introduction of amino functionality. Further, the H/C ratio was the same, while O/C and (N + O)/C ratios were increased, confirming that the polarity of the modified adsorbent was decreased, while its hydrophobicity was increased. The adsorption kinetics suggested that capturing Cd(ii) over the surfaces of both pristine and modified adsorbents was spontaneous and fast, as equilibrium was attained in 4 h. The IPD model realized adsorption through two steps. The first step, named the liquid film diffusion step, was controlled by the migration of Cd to the surface of the adsorbent, while the second step, named the IPD step, was dominated by the migration of Cd from the surface to the inner pores of the adsorbent, as shown in Fig. 5f. The applications of biochar-based adsorbents are summarized in Table 1.

**Table 1:** Adsorptive removal of toxic materials using biochar-based adsorbents

Biomass type	Adsorbent	Method of preparation	Temperature/duration	Target pollutant	Amount of pollutant removed	Ref.
Marine green alga enteromorpha flexuosa	Biochar activated by natural kaolinitic clay composite	Pyrolysis and co-precipitation	600 °C/3 h	Norfloxacin and crystal violet dye	192.80 mg per g norfloxacin and 281.24 mg per g crystal violet dye	75
Peanut shells	Pure biochar	Pyrolysis	500 °C/4 h	Ciprofloxacin and Pb	58.78 mg per g CIP and 103.66 mg per g Pb	76
Bamboo chopsticks	Pure biochar	Pyrolysis	300–800 °C/2 h	Norfloxacin	99.71%	77
Caragana korshinskii	BiVO_4_/biochar	Pyrolysis/hydrothermal	650 °C/3 h	Rhodamine B, tetracycline, norfloxacin, and chloramphenicol	73.50% rhodamine and more than 73.50% tetracycline	78
Water hyacinth	Biochar activated by Na_2_CO_3_/K_2_CO_3_	Pyrolysis/hydrothermal	600–800 °C/2 h	Phenol	Adsorption (121.3 mg g^−1^)/catalytic (99.84%)	79
Pomegranate peels	ZnO nano-flowers/biochar nanocomposite	Pyrolysis/hydrothermal	600 °C/3 h	Phenol, methylene blue and rhodamine B	91% phenol, 94% methylene blue and 90% rhodamine B	80
Wheat straw	Mn/Mn_3_O_4_ modified biochar	Pyrolysis/hydrothermal	500 °C	Cephalexin	80% in 28 days	81
Sludge from municipal wastes	Oxalic acid modified biochar	Pyrolysis	600 °C/2 h	Methyl orange and pyrene	100% in 80 min	82
Wood chip	Fe/Ti biochar composite	Pyrolysis	300–600 °C/2 h	Ciprofloxacin and norfloxacin	Ciprofloxacin (88.4%) and norfloxacin (88.0%)	83
Coconut fiber	γ-Fe_2_O_3_/biochar composite	Ball-milling and phosphomolybdic acid and Fe(NO_3_)_3_ co-promoted pyrolysis	300–400 °C/1 h	Tetracycline	631.53 mg g^−1^	84
Palm oil mill sludge	Pure biochar	Pyrolysis	200–600 °C/1.5 h	Cu and Cd	Cu (48.8 mg g^−1^) and Cd (46.2 mg g^−1^)	85
Date seed	Pure biochar	Pyrolysis	550 °C/3 h	Pb, Cu and Ni	Pb (0.911 mmol g^−1^), Cu (0.911 mmol g^−1^), and Ni (0.692 mmol g^−1^)	86
Watermelon rinds	Pure biochar	Pyrolysis	500 °C/1 h	Ti(i)	178.4 mg g^−1^	87
Anaerobically digested sludge	Pure biochar	Pyrolysis	600 °C/4 h	Pb and Cd	0.75 mmol g^−1^ of Pb(ii) and 0.55 mmol per g Cd(ii)	88
Soft wood pine	Urea-functionalized biochar	Ultrasound-assisted preparation	—	Ni	>99%	89
Straws of oil seed rape	Pure biochar	Pyrolysis	550–700 °C	Cd	—	90
Wood, chicken manure, and food waste	KOH-activated biochar	Pyrolysis	—	Radioactive Cs and Sr	Cs (62.7 mg g^−1^) and Sr (43.0 mg g^−1^)	91
Waste watermelon rind	Magnetized biochar	Pyrolysis/hydrothermal	500 °C/1 h	U(vi)	323.56 mg g^−1^	92
Oil tea shell	ZnCl_2_ impregnated biochar	Pyrolysis/hydrothermal	—	Nitrate	15.6 mg g^−1^	93
Bamboo waste	Activated charcoal/biochar	Pyrolysis	600–900 °C/4 h	Palladium(ii)	80.32–60.16% and metal uptake of 6.69–50.13 mg g^−1^	94
Rice-straw	Ferric chloride-treated biochar	Pyrolysis	450 °C/4 h	Hg(ii)	85.8 mg g^−1^ or 94.91%	95
Pomelo fruit peels	Pure biochar	Pyrolysis	500 °C/45 min	Pb(ii)	92.13 mg g^−1^	96
Spent coffee ground biochar	Bi-impregnated biochar	Pyrolysis	600 °C/1 h	Radioactive iodine	253.71 µg g^−1^	97
Cabbage leaves	Anthocyanin and kaolinite modified biochar	Pyrolysis	350 °C/4 h	Pb and Cu	188.67 mg per g Pb and 48.07 mg per g Cu	98
Coffee waste	NaOH/H_2_O_2_ modified biochar	Pyrolysis/post modification	500 °C/2 h	Sr(ii)	10.91 mg g^−1^	99
Buckwheat hull-derived biochar	Biochar immobilized in alginate beads	Pyrolysis	450–900 °C/1 h	Co	24.0 mg g^−1^	100
Fish scales	Multi-heteroatom self-doped microporous biochar	Pyrolysis	600 °C/1 h	U(vi)	142.06 mg g^−1^	101
Bamboo waste	Fe_3_O_4_ immobilized biochar	Pyrolysis/hydrothermal	—	U(vi)	70.45 mg g^−1^	102
Pomelo peels	Phosphorus-doped biochar	Pyrolysis	750 °C/1.5 h	Uranium	603 mg g^−1^	103
Dictyophora indusiata	Biochar-supported sulfurized zero-valent iron	Hydrothermal and *in situ* growth method	—	U(vi)	300.2 mg g^−1^	104
Sewage sludge	FeCl_3_-activated biochar	Pyrolysis/hydrothermal	600 °C/2 h	As(iii) and Cr(vi)	47.46 mg per g Cr(vi) and 5.0 mg per g As(iii)	105
Orange waste	Biochar-magnesium silicate modified biochar	Pyrolysis/hydrothermal	650 °C/2 h	U(vi)	352.6 mg g^−1^	106
Pine sawdust	Microwave-assisted one-pot prepared ZnO decorated biochar	Pyrolysis	600 °C/30 min	Levofloxacin and Cr(vi)	193.42 mg per g levofloxacin and 21.03 mg per g Cr(vi)	107
Peanut shells	ZnO/N and O-doped biochar nanocomposite	Solvothermal process coupled with calcination	600–800 °C	Methylene blue, methyl violet, methyl orange, and tetracycline	98.71% methylene blue in 100%	108
Fecal sludge	TiO_2_ and ZnO impregnated biochar	Pyrolysis	600 °C/1 h	Malachite green	98%	109

#### 4.1.4 Adsorptive removal of radioactive materials

**Fig. 6:**
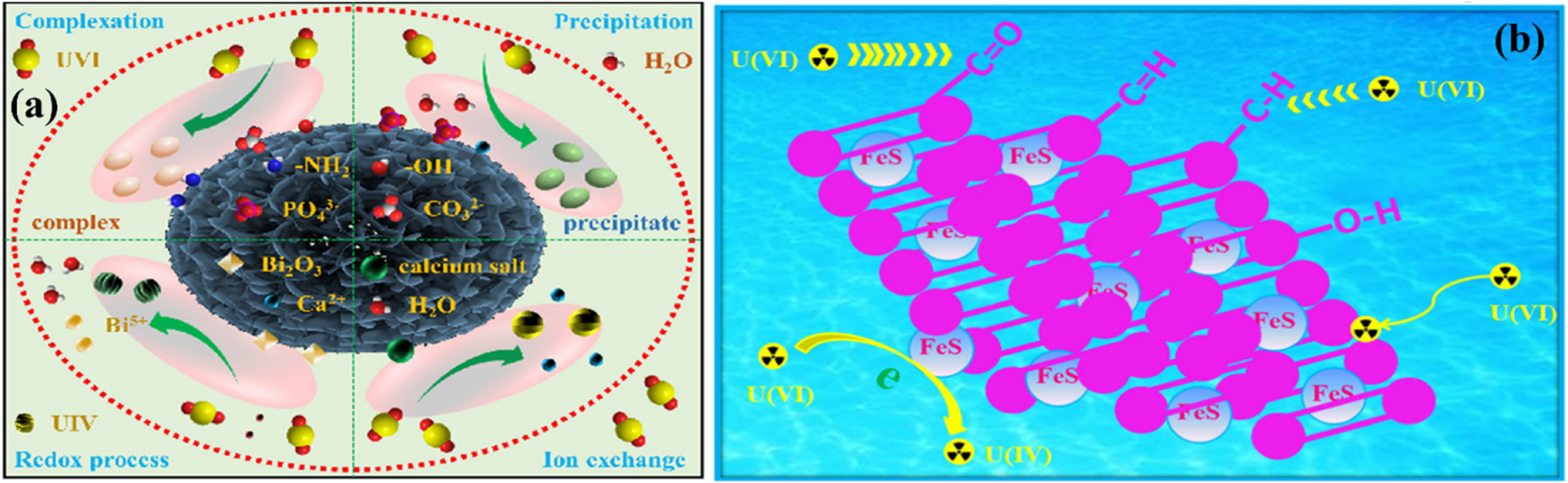
Adsorption of UO_2_^2+^ over modified BO/HOC biochar (a), figure adapted/reproduced from ref. 111 with permission from Elsevier [(license number: 6145871187390) copyright 2025], and that of U(vi) over CFS/BC (b). Figure adapted/reproduced from ref. 112 with permission from Elsevier [(license number: 6145871303708) copyright 2025].

Radioactive materials play a crucial role in human society. They are frequently used in nuclear power plants to generate electricity and cope with carbon emissions. They have numerous applications in the medical and aeronautical sciences. However, they are very toxic and extremely detrimental to animals, plants, and the environment. Extreme exposure to radioactive materials, like uranium, may induce inflammation, genotoxicity, skin and circulatory issues, leukemia, and cancer. Uranium is generally released to natural water bodies in the hexavalent uranyl (UO_2_^2+^) state from the mining, smelting, and processing uranium salts, electronics, ammunition, and fertilizer industries.^110^ Considering the high environmental toxicity of uranium, it is very important to separate it from wastewater to prevent its hazardous applications. Liao *et al.* prepared biochar from horse manure, followed by treatment with H_2_O_2_. The H_2_O_2_-treated biochar (HOC) was further treated to obtain Bi_2_O_3_-modified (BO/HOC) biochar for the adsorptive removal of uranium.^111^ The effect of solution pH was significant in the removal of uranium. The modified BO/HOC biochar showed remarkable activity in the removal of uranium at the optimum pH value. When the pH of the solution was less than 4, uranium existed as the UO_2_^2+^ ion. It was found that the surfaces of the pristine and modified BO/HOC biochar were negatively and positively charged, respectively, when pH_pzc_ > pH > pH_pzc_. When the pH was less than 4, both pristine and modified BO/HOC biochar removed a significant amount of uranium due to the electrostatic forces of attraction between the positive UO_2_^2+^ ion and negative adsorbents. When pH was increased beyond 4.0, the removal activity was significantly reduced due to the formation of hydroxyl complexes, such as (UO_2_)_3_(OH)_7_^−^, UO_2_(OH)_3_^−^, (UO_2_)_4_(OH)_7_^+^ and (UO_2_)_3_(OH)_5_^+^. The formation of these negatively charged species notably reduced the electrostatic interaction between the UO_2_^2+^ ion and adsorbents to retard adsorption. From the SEM images, a uniform distribution of particles indicated that uranium accumulated on the surface of the modified BO/HOC biochar. A change in the XPS peak of P 2p after adsorption suggested complex formation between uranium and PO_4_^3−^. Similarly, a reduction in the Ca 2p peak intensity confirmed the ion exchange mechanism. Thus, adsorption was synergistically ascribed to electrostatic interactions; generation of different species by the chemical reaction of UO_2_^2+^ ions with –NH_2_, CO_3_^2−^, and ^−^OH; precipitation of UO_2_^2+^ with PO_4_^3−^; ion exchange with calcium salt; and redox conversion of UO_2_^2+^ ion to UO_2_, as shown in Fig. 6a.

FeSO_4_-modified biochar was prepared using peanut shells by Chen *et al.* for the eradication of uranium from wastewater.^112^ The SEM images indicated that irregular snowflakes, like nanoparticles of FeS, were agglomerated on the surface of biochar. The rate of adsorption was notably boosted when the pH was changed from 2 to 4. However, a further increase in pH from 6 to 10 gradually decreased the eradication efficiency of uranium. This increase and decrease in removal efficiency were related to the different electrostatic interactions between the adsorbent and adsorbate. Different forms of uranium (UO_2_^++^, UO_2_(OH)^+^, U_2_O_4_(OH)_2_^++^, UO_2_(OH)_2_, U_3_O_6_(OH)_7_^−^, UO_2_(OH)_3_^−^ and UO_2_(OH)_4_^2−^) are found under different pH conditions. The negatively charged species existed at a pH value of 6.2, showing significant repulsion with the surface of the adsorbent to retard the removal efficiency of uranium. Electrostatic interaction, complex formation, and precipitation processes synergistically removed uranium from wastewater, as shown in Fig. 6b.

#### 4.1.5 Adsorptive removal of anions

Despite organic dyes, antibiotics and heavy metals, many anions, such as phosphate, nitrate, sulphate, halides, and bicarbonate, are frequently found in water and disturb the natural environment if their concentration is beyond the limited permissible concentration. Among these anions, phosphate and nitrate have enormous applications, and the so-called eutrophication phenomenon is attributed to their doses in natural water.^113^ Although natural processes contribute a major portion of these anions to the environment, anthropogenic contributions from fertilizer industries are seriously intensifying their concentrations in different water bodies.^114^

##### 4.1.5.1 Adsorptive removal of nitrate

**Fig. 7:**
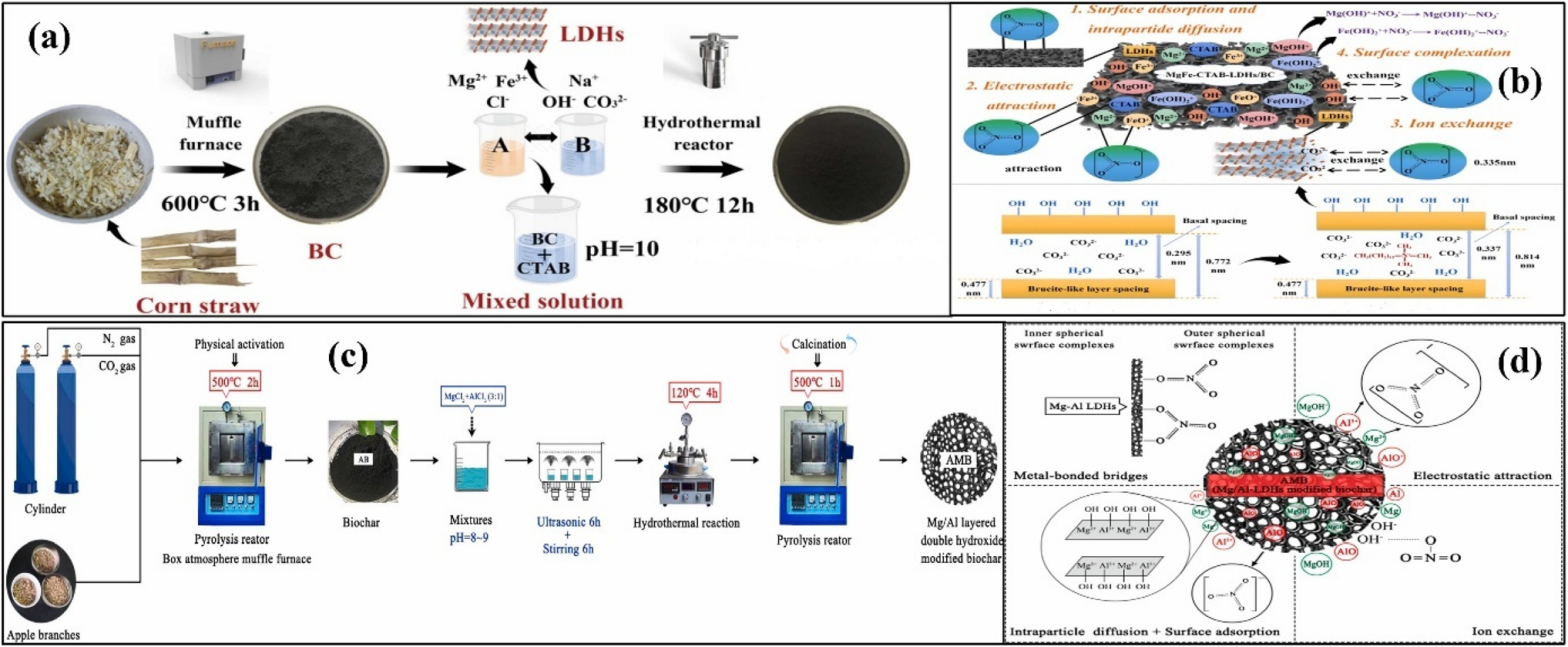
Synthetic procedure for (MgFe/CTAB)BC (a) and schematic for the adsorption of nitrate by Mg and Fe double hydroxides and (MgFe/CTAB)BC (b), figure adapted/reproduced from ref. 118 with permission from Elsevier [(license number: 6145911503803) copyright 2025]. Synthesis (c) and adsorption mechanisms of nitrate over AMBC (d). Figure adapted/reproduced from ref. 119 with permission from Elsevier [(license number: 6145920215887) copyright 2025].

Both nitrogen and phosphorus are extremely vital elements found in animal and plant cells. Nitrogen is a fundamental and integral constituent of all types of proteins, while phosphorus is found in the DNA and RNA of the cells to transmit hereditary character and energy.^115^ However, their presence in natural water beyond a certain limit causes considerable ecological concerns. Many adsorbents have been used to remove and recycle nitrate and phosphate for useful applications. However, the removal efficiencies of single pristine adsorbents are not satisfactory.^116,117^ The synergetic effect of two or more substances in the removal of hazardous materials is significant compared to pristine materials. To integrate the properties of the different substances, Wang *et al.* applied corn straw to prepare MgFe-loaded and CTAB-modified biochar, as shown in Fig. 7a.^118^ The presence of biochar significantly improved de-agglomeration and active sites, while the arrival of CTAB brought a positive charge and improved interlayer spacing in the hydroxides. A decrease in adsorption was observed when the operational pH was increased owing to the excessive occupation of the active sites by the OH^−^ ions, while an increased adsorption was realized at lower pH values due to the attractive electrostatic forces. The adsorption of nitrate was further monitored by considering surface adsorption, complexation, pore diffusion, electrostatic interaction, and exchange of nitrate ions with OH^−^ groups. The linear fitting of the SSO kinetics demonstrated that the adsorption was mainly contributed by the chemical adsorption of nitrate, as supported by the Langmuir model, indicating monolayer adsorption. The adsorptive removal of nitrate was also performed in the presence of hydrogen carbonate, carbonate, phosphate, sulphate, and chloride at the same concentration as that of the target nitrate ions. All these interfering anions exhibited different inhibitory effects, with the highest effect powered by phosphate and the lowest by hydrogen carbonate and chloride. At a lower temperature, adsorption was reduced in magnitude due to the slow movement of nitrate ions, as shown in Fig. 7b.

Direct pyrolysis normally introduces negatively charged oxygen containing functional groups, which drastically reduces the anion exchange capability of the prepared adsorbents. Therefore, the physical and chemical modification/activation of biochar-based adsorbents has attracted significant attention for the removal of hazardous materials. Physical modification is achieved in two steps: carbonization and activation. The process is initiated by the carbonization of biomass, followed by activation with either CO_2_ or H_2_O. For example, Wang *et al.* obtained biochar from apple-based biomass in an O_2_-free atmosphere, followed by activation with CO_2_.^119^ The prepared biochar was further modified with AlMg-based double hydroxides to form an AMBC for the adsorption of nitrate, as shown in Fig. 7c. Under acidic conditions, the adsorbent was positively charged due to an elevated number of H^+^ ions. The electrostatic interaction between the positive adsorbent and negative nitrate ions significantly accelerated the adsorption process. As the pH increased, the surface of the adsorbent gradually attained a negative charge to generate electrostatic repulsion between the adsorbent and nitrate ions. The process comprised two steps: initial fast and later sluggish. This was ascribed to the more available sites for adsorption in the initial stages of adsorption. The ions migrated to the inner pores and then through the middle into the small pores. The SSO showed a larger *R*^2^, confirming that nitrate ions were physically and chemically adsorbed through the IPD and liquid film diffusion mechanisms. Chemical adsorption was followed by studying the FTIR spectra of the adsorbent before and after the adsorption of the ions. The spectra demonstrated that the peaks due to –OH and –CO_2_H were faded owing to the exchange of nitrate ions. Similarly, vibrational peaks related to C=O drastically faded, suggesting that AlMg double hydroxides contributed to the removal of ions. The FTIR peak related to aromatic cycles was significantly changed, indicating that their π-electrons contributed significantly to the formation of a stable complex with nitrate ions, as shown in Fig. 7d.

##### 4.1.5.2 Adsorptive removal of phosphate

**Fig. 8:**
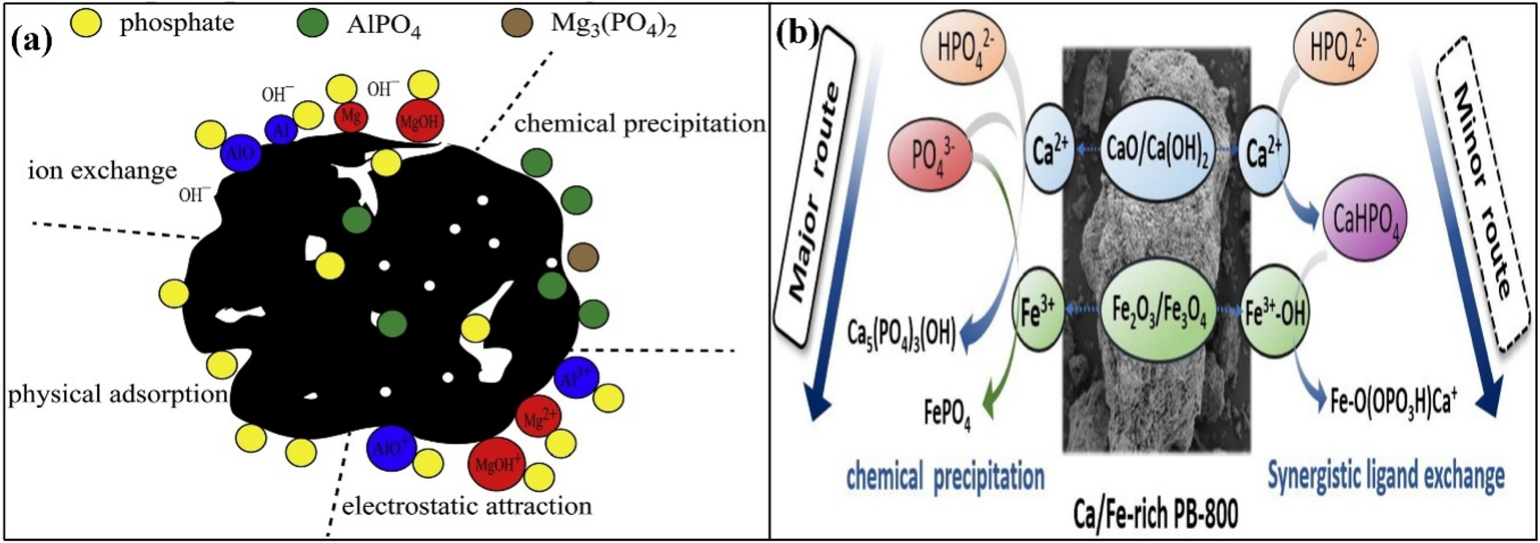
Schematic for phosphate removal by MgAl/BC (a) figure adapted/reproduced from ref. 125 with permission from Elsevier [(license number: 6145921355116) copyright 2025], schematic phosphate removal by PB-800 (b). Figure adapted/reproduced from ref. 126 with permission from Elsevier [(license number: 6145930122452) copyright 2025].

Phosphorus is an important element in the constitution of animal bones. Like nitrate, it is also an important element that influences the growth and development of plants.^120–122^ It is also present in appreciable quantities in natural water bodies and causes ecological issues, like eutrophication of natural ponds. The sources of phosphorus in nature are limited, and it is believed that all its sources will be exhausted in the coming 100 years. Therefore, to reserve the sources of phosphorus for the coming generation and to meet future phosphate-based fertilizer demand, the removal of phosphate from water is an important task in ecological environmental management.^123,124^ For the effective removal of phosphate, Zheng *et al.* prepared Mg- and Al-loaded biochar from wheat straw.^125^ Interestingly, all the prepared adsorbents showed pointedly extended phosphate removal efficiencies ascribed to the availability of many active sites and operative functionalities. The role of the solution pH was magnificent. As the pH changed from acidic to basic, the pristine biochar showed a significant reduction in the phosphate removal efficiency, while the modified biochar imparted the highest activities in the acidic environment and relatively low activities in the basic environment. The FTIR and XRD studies of the adsorbents before and after adsorption revealed that surface adsorption, ion exchange, and electrostatic interactions were involved in the removal of phosphate ions from water, as shown in Fig. 8a.

Yu *et al.* prepared metal-modified biochar-assisted adsorbents for the exclusion of phosphate from water. Sludge from a paper mill was heated at different temperatures in a tube furnace under a continuous flow of CO_2_.^126^ The morphological images demonstrated that no particles were found on the surface of the adsorbent prepared at a low temperature. However, as the temperature increased, aggregates of crystalline nanoparticles were observed on the surface of the adsorbents. The elemental analysis confirmed that these nanoparticles were mainly composed of iron and calcium oxides. The adsorption kinetics conveyed that the data were well defined by the SSO kinetics, as the value of *R*^2^ was greater than the SFO kinetics, demonstrating that adsorption was controlled by chemosorption owing to the chemical interaction of phosphate with different adsorbents. From the obtained data, it was realized that the elimination of phosphate was initiated by precipitation induced by Ca. At elevated temperatures, oxides and hydroxides of Ca were hydrolysed to form Ca^2+^ and CaOH^+^ along with the liberation of OH^−^. The formed Ca^2+^ and OH^−^ reacted with HPO_4_^2−^ and PO_4_^3−^ to initiate the precipitation of phosphate in the form of Ca_5_(PO_4_)_3_(OH) and CaHPO_4_. Phosphate was also precipitated by the OH^−^ groups of iron oxide with the aid of inner–sphere complex formation and FeHPO_4_ and FePO_4_ formation. Thus, the synergetic effects of both Ca and Fe resulted in the removal of phosphate through the collective efforts of precipitation, ion exchange, complexation, and physical adsorption. However, precipitation in the form of Ca_5_(PO_4_)_3_(OH) and FePO_4_ was the major contributor to the adsorptive removal of phosphate, as shown in Fig. 8b.

### 4.2 Biochar as a photocatalyst

Biochar also plays a crucial role in photocatalysis to remove toxins and generate energy at a low cost. Owing to its porous nature, blackish color and excellent graphitic nature, biochar plays a very commanding role in photocatalysis.^127^ Here, we debate the significance of biochar in photocatalysis.

#### 4.2.1 Light absorption

Like natural photosynthesis, artificial photosynthesis also requires the absorption of solar light to propagate redox reactions with the aid of photogenerated charges.^128^ Through the courtesy of suitable solar photons, charge excitation and deexcitation take place in semiconductor materials. The photophysical excitement of semiconductors results in the formation of a highly unstable state of negatively charged species in the conduction bands (CBs) and a corresponding positively charged state in the valence bands (VBs).^129,130^ The activity of the excited electrons is measured in terms of their reductive energy, while the efficiency of the holes is directly related to their oxidative energy. The thermodynamic energies of both reduction and oxidation in turn depend on the energy of the striking photons, causing momentary polarization in the working materials.^131^ Since UV light is more energetic than visible light; therefore, absorption in this region produces highly energetic species with large thermodynamic oxidative and reductive energies for the propagation of redox reactions. However, the solar spectrum carries only about 5% UV photons compared to the wider range of visible light.^132,133^ Therefore, researchers are more interested in the construction of materials that are active under visible light. Biochar is a special material with a black or brown color that carries a high concentration of carbon elements. It also possesses certain nitrogen functionalities. These characteristics are exceptionally helpful in assisting in the absorption of solar light in the visible range. Particularly, the interlayered spaces in layered semiconductor materials are reduced when biochar is added, which helps to improve light absorption. Carbon-rich materials are crucial in augmenting light absorption. Therefore, integration of the base semiconducting materials with biochar assists in the absorption of visible light to generate more excited charges for the propagation of light-assisted redox reactions.^134,135^

#### 4.2.2 Charge separation

The efficiency of light-assisted photocatalytic redox reactions depends on the generation of excited charges with high thermodynamic energies and their complete separation for a longer time. If the excited charges are not separated for enough time, they recombine with each other to reduce the efficiency of the redox process.^136^ Biochar also plays an exceptional role in the separation of excited charges. The outstanding carbonaceous nature of biochar is believed to separate the excited charges effectively because many of these materials have provided the best results. Porous biochar possesses excellent channels for the migration and separation of workable charges.^137,138^ It provides reduced diffusion dimensions for the migration of excited charges towards their respective oxidation and reduction centers. The porous structure of biochar functions as a contrary quantum dot, which highly disperses the working oxidation and reduction centers to encourage the efficient separation of excited charges. The presence of nitrogen and oxygen functionality easily accepts the arrival of excited electrons owing to their high electronegativities and excellent deriving energies to assist in the migration and separation of excited charges for efficient photocatalysis.^139,140^

#### 4.2.3 Adsorption of reactive species

The excited electrons and holes on arrival at the surface of the photocatalysts react with adsorbed species to realize their effective reduction and oxidation.^141^ Therefore, the adsorption of reactive species plays a vital role in the efficiency of photocatalysis. Biochar provides a highly porous and large surface area with exceptional pore volumes and active sites for the adsorption of target species.^142^ When the reactive species are properly adsorbed on the surface of photocatalysts, their activation energies are largely reduced depending on whether adsorption is chemical or physical. A reduction in activation energy is directly associated with the breaking of bonds in the target pollutants to form new, less hazardous products. The polarity of biochar due to oxygen and nitrogen functionality and hydrophilicity to –OH functionality preferentially attracts polar and charged species towards its surface for effective adsorption and photocatalysis.^143^

### 4.3 Biochar-based photocatalysis

Photocatalysis has been exploited exclusively in the last few decades to deal with hazardous pollutants and minimize their toxic effects. Many photocatalysts have been utilized with exceptional activities. However, pristine photocatalysts have many inherited shortcomings, such as low surface areas, high charge recombination of the excited charges and low thermal and charge conductivities. Biochar-based photocatalysts generally possess large surface areas and high charge conductivities to overcome the inherited drawbacks to some extent and improve the rate of slow kinetics.^144^ Here, we discuss the significance of biochar-based photocatalysts in the removal of toxic substances.

#### 4.3.1 Photocatalytic removal of pollutants

**Fig. 9:**
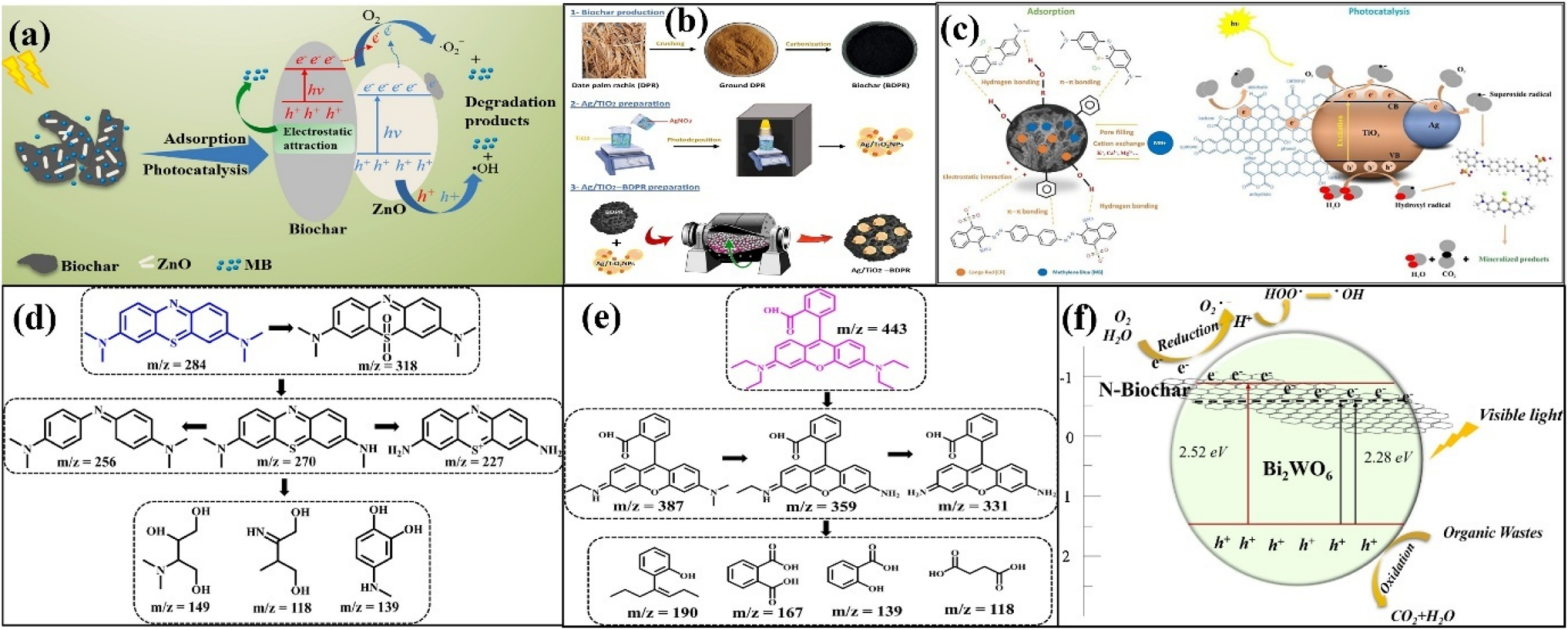
Oxidation of MB by ZBP nanocomposites under light interaction (a), figure adapted/reproduced from ref. 150 with permission from Elsevier [(license number: 6145930382738) copyright 2025]. Synthesis of the Ag-doped TiO_2_ coupled biochar (b), and schematic for the decomposition of dyes (c), figure adapted/reproduced from ref. 153 with permission from Elsevier [(license number: 6145930579118) copyright 2025]. Degradation pathway of MB (d) and decomposition of RhB over BC/2ZIS/WO_3_ (e), figure adapted/reproduced from ref. 155 with permission from Elsevier [(license number: 6145930841962) copyright 2025]. Schematic of the charge separation in BW/N-B (f). Figure adapted/reproduced from ref. 158 with permission from Elsevier [(license number: 6145931067101) copyright 2025].

The discharge of many hazardous substances from manufacturing industries has caused great concern about environmental pollution in the past few decades. All segments of the environment (soil, water, and air) have been loaded with enormous amounts of toxic and hazardous materials.^145–147^ The polluted environment has badly disturbed the natural ecosystem belonging to plants and animals. Human beings suffer severely and many fatal diseases, including cancer, lung problems, skin diseases, eye irritations, and mental issues, have been reported throughout the world. To detoxify the polluted environment, many techniques have been applied, including adsorption, coagulation, filtration, and advanced oxidation, to eradicate toxic substances from every segment of the environment.^148^ However, none of these techniques has achieved enough popularity due to their low pollution removal efficiencies. Photocatalysis is believed to solve energy and environmental issues efficiently with minimum energy utilization and labor requirements. The effective application of photocatalysis requires smart semiconductors/photocatalysts with proper utilization of solar energy, effective separation of charges and large amounts of active sites for adsorption and desorption of the target materials.^149^ Biochar-based photocatalysts have attracted significant attention due to their graphitic and porous nature, which provides excellent conductivity and many sites for the frequent adsorption and oxidation of hazardous materials. Biomass from bamboo was heated at 600 °C for 120 min in a furnace by Yu *et al.*, followed by coupling with ZnO nanoparticles to form ZnO/biochar-based photocatalyst (ZBP) for the photocatalytic oxidation of MB.^150,151^ SEM and TEM images confirmed the distribution of small rod-like ZnO particles. N_2_ adsorption/desorption confirmed the presence of macro and micropores with a trace amount of mesopores, which significantly improved the adsorption of methylene blue for photocatalytic degradation. The biochar significantly improved the charge conductivity in the nanocomposites and the dispersion and stabilization of the ZnO particles. Under irradiation conditions, the adsorbed methylene blue was degraded by the reactive radicals produced. Photocatalytic degradation was performed by the ZnO nanoparticles, while biochar helped adsorb the target pollutant and conduct the photogenerated charges effectively. The photogenerated electrons in the CB of ZnO were advanced to the adsorbed O_2_ molecules through the coupled biochar for the generation of super oxide anions, while holes in the VB of ZnO produced ˙OH free radicals by the reaction with adsorbed water to oxidize methylene blue effectively, as shown in Fig. 9a.

The functionalization of biochar-based photocatalysts for useful applications has recently been a flashpoint.^152^ The fabrication of multifunctional biochar-based photocatalysts for environmental and energy issues has been provoked throughout the globe. Chakhtouna *et al.* functionalized date palm-based biochar with Ag-doped TiO_2_ for the oxidation of organic dyes and the inactivation of bacteria, as shown in Fig. 9b.^153^ SEM pictures revealed a uniform distribution of Ag-doped TiO_2_ on the surface as well as in the pores of biochar. The light absorption capacity of TiO_2_ was significantly improved with the introduction of Ag nanoparticles with surface plasmon resonance property.^154^ Both adsorption and photocatalytic experiments were conducted using methylene blue and Congo red as model pollutants. When the photocatalysts were irradiated under UV light, the complete oxidation of methylene blue (at pH = 6) and Congo red (at pH = 4) was realized in 75 and 150 min, respectively. The enhanced decomposition of both dyes was attributed to the collective role played by biochar and surface plasmon resonance Ag-doped TiO_2_. The photocatalytic decomposition of both dyes with pristine biochar did not enhance UV light, demonstrating its non-photocatalytic nature. A schematic, as shown in Fig. 9c, has been proposed to demonstrate the photocatalytic decomposition of both dyes over the nanocomposite and is governed by the following three steps.

(a) Diffusion of dye particles from the solution to the surface of the nanocomposite.

(b) Adsorption of dye particles over the nanocomposite through various interactions.

(c) Redox reactions to decompose the adsorbed dye particles.

Under UV light irradiation, excited electrons in the CB of the nanocomposite reduced O_2_ to ˙O_2_^−^, while holes in the VB oxidized H_2_O to hydroxyl free radicals. The generated radicals initiated the decomposition of the target dyes by opening the benzene rings, cleaving the chemical bonds and generating different intermediates. The formed intermediates were completely converted into CO_2_ and H_2_O through subsequent attacks on the formed radicals. The arrival of biochar and Ag nanoparticles significantly improved the photocatalytic performance of TiO_2_ by improving charge conductivity, enhancing carrier separation, and extending light absorption for highly speedy photocatalysis.

The formation of a well-constructed heterojunction between two semiconductors, followed by incorporation in biochar, expands charge separation and light absorption due to the synergetic effect of the heterojunction and graphitic structure of biochar. Cheng *et al.* established a suitable heterojunction between metal sulfides and WO_3_, followed by incorporation into biochar.^155^ The SEM images suggested a porous defective structure of biochar, which further developed when treated with KOH and Ca(OH)_2_. The nanocomposite provided a lamellar structure due to its layered hexagonal morphology. The dye removal efficiency of pristine WO_3_ was poor due to the high recombination efficiency of the excited charges. However, charge separation was significantly improved after the introduction of biochar due to the heterojunction formation and graphitic nature of biochar. The intermediates of both dyes were measured, as shown in Fig. 9d and e. The reactive species produced during photocatalysis attacked the complex structures of the two dyes and formed simple compounds by ring opening, hydroxylation, demethylation, and deamination.

For rapid and fast photocatalysis, the separation of excited charges is absolutely essential. However, in many pristine photocatalysts, the rate of excited charge recombination is very fast, and very few electrons/holes are present for the oxidation reactions, thereby drastically decreasing the rate of photocatalysis even though many excited charges are produced during a given time. However, the incorporation of biochar significantly increases the rate of exciton separation due to its graphitic nature.^156,157^ Therefore, many authors have used biochar from different sources to couple with semiconductor photocatalysts to improve highly speedy photocatalysis. Wang *et al.* coupled N-doped biochar with Bi_2_WO_6_ for the eradication of rhodamine B to form BWO/NBC.^158^ The SEM images confirmed the distribution of small BWO particles on the surface of the large 2D NBC nanosheets. Interestingly, the BWO/NBC imparted high degradation activity compared to pristine BWO. It was learned that the decomposition of RhB was not significantly affected by dye adsorption. Therefore, it was suggested that the decomposition was mainly attributed to the photocatalytic function of the fabricated samples. Measurement of the rhodamine B concentration after a regular interval suggested that the maximum absorption peak was not changed during adsorption measurement, while a definite hypsochromic shift in the absorption peak during photocatalysis indicated that chemical changes were taking place in the structure of rhodamine B. After 90 min, the hypsochromic shift was minor, indicating that rhodamine B degraded in the aqueous solution. The degradation mechanism is illustrated in Fig. 9f. Obviously, after light irradiation, excited charges are produced in the form of electrons and holes in the CB and VB of the photocatalyst, respectively. The N-doped biochar significantly improves charge separation and light absorption due to its graphitic nature and N-dopants. Thus, the recombination of excited charges is restricted, and more charges are available for the decomposition of pollutants. The applications of biochar-based photocatalysts/catalysts are summarized in Table 2.

**Table 2:** Photocatalytic/catalytic removal of pollutants using biochar-based materials

Biomass type	Photocatalyst	Method of preparation	Temperature/duration	Target pollutant	Amount of pollutant removed	Ref.
Cinnamon barks	Biochar/TiO_2_/CoFe_2_O_4_	Pyrolysis/hydrothermal	400 °C/1 h	Chlorophenol	92%	159
Walnut shell	Biochar supported TiO_2_/g-C_3_N_4_	Microwave pyrolysis	—	Congo red	60% in 3 h	160
Walnut shells	Biochar/Fe_3_O_4_/CuO composite	Pyrolysis/co-precipitation	600 °C/2 h	Norfloxacin	94.5% 180 min	161
Fly ash and rice husk	Cobalt supported on silica-composited biochar	Pyrolysis	700 °C/5 h	Phenol	98%	162
Jasmine raw materials from the jasmine tea processing factory	Pure biochar	Pyrolysis	400–700 °C/2 h	Tetracycline	90%	163
Pine chips	Ura treated biochar	Pyrolysis	600 °C/2 h	Metronidazole	99.6%	164
	Zinc stannate/biochar composite	Co-calcination/hydrothermal	450 °C/4 h	Rhodamine B	99.4% in 45 min	165
Waste cellulose	Co–Fe oxide/biochar composite	Microwave-assisted carbonization	500 °C/4 h	4-Nitrophenol	99.0%	166
Crude peach gum	Multivalent iron-based biochar composite	Pyrolysis	800 °C/2 h	Methylene blue, methyl violet and coccine	100% in 16 min of methylene blue	167
Poplar powder	PbMoO_4_/biochar/Fe_3_O_4_ composite	Pyrolysis/co-precipitation	500 °C/2 h	Tetracycline	89% in 1 h	168
Red mud and shaddock peel	Fe-containing red mud biochar	Co-pyrolysis	800 °C/2 h	Acid orange 7	87%	169
Cotton fibers	Bi_2_WO_6_/biochar	Hydrothermal synthesis	170 °C/24 h	Rhodamine B and tetracycline	99.9% rhodamine B and 96.8% tetracycline	170
Municipal sludge	Pure biochar	Pyrolysis	700 °C/2.5 h	Sulfadiazine	100% in 2 h	171
Camellia oleifera shell	FeS/Fe_3_O_4_ co-modified biochar	Pyrolysis	900 °C/2 h	Quinclorac	100%	172
Walnut shell	N-doped TiO_2_/biochar	Pyrolysis/co-precipitation	700 °C/2 h	Methyl orange	97.6%	173
Alkaline lignin	Pure biochar	Pyrolysis	350–750 °C/1.5 h	Bisphenol A, ascorbic acid, heteroauxin and cysteine	77.2% bisphenol A	174
Chenopodium ambrosioides leaf extract	Ag@biochar	Pyrolysis/green synthesis	550 °C/3 h	Methylene blue	88.4%	175
Straw	Mn/N/S co-doped modified biochar	Pyrolysis	500 °C/2 h	Ciprofloxacin	99% in 1 h	176
Seaweed (sargassum and enteromorpha)		Pyrolysis	800 °C/20 min	Hg	91.34%, and 88.26% under 4% H_2_O_2_ and 120 °C	177
Sewage sludge	Cu-loaded biochar	Pyrolysis	450–750 °C/1.5 h	Bisphenol A	100 mg L^−1^ in 30 min	178
Industrial lignin	MnFe_2_O_4_/biochar composite	Pyrolysis/co-precipitation	225 °C/10 h	Oxytetracycline	90% degradation in 30 min	179
Sugarcane bagasse	H_2_–TiO_2_/biochar composite	Pyrolysis/hydrothermal	400 °C/3 h	Enrofloxacin	95.6% in 3 h	180
Rice straw	Biochar-loaded metal oxides (Fe/Co/Cu)	Pyrolysis	800 °C/2 h	Ciprofloxacin	99.99%	181
Sawdust powder	Fe_3_O_4_-loaded biochar	Pyrolysis	—	Acid orange 7	90% in 1h	182
Sewage sludge	Biochar-loaded nZVI	Pyrolysis	400 °C	Reactive blue 4	99.56%	183
Waste from palm oil fruit	Biochar-supported nano bismuth ferrite composite	Pyrolysis/hydrothermal	700 °C/1 h	Ciprofloxacin	77.08% in 2 h under direct sun light	184
Lotus leaves	Nitrogen self-doped biochar	Pyrolysis	700–900 °C/2 h	Orange II	97.07%	185
Animal-derived biochar	Pure biochar	Pyrolysis	500 °C/4 h	Pb(ii), Cd(ii), Cu(ii), and Hg(ii)	—	186
Calotropis gigantea leaves	Biochar-ZnO composite	Pyrolysis/precipitation	520 °C/4 h	Ciprofloxacin	80% in 4 h	187
Rice husk	TiO_2_/rice husk biochar	Pyrolysis/hydrothermal	—	Bisphenol A	97.6% in 1 h	188
Sunflower straw powder	Biochar-based supramolecular self-assembled g-C_3_N_4_	Pyrolysis	500 °C/3 h	Phenanthrene	76.72%	189
Wood flour	g-C_3_N_4_/biochar/BiVO_4_ photocatalyst	Pyrolysis/hydrothermal	540 °C/5 h	Rhodamine B	98.2% in 30 min	190
Soybean stalks	TiO_2_/biochar composite	Pyrolysis/soak calcination method	500 °C/4 h	Phenol	77.7%	191
Tannery sludge	Biochar and zinc oxide nanoparticles	Hydrothermal	180 °C/12 h	Bisphenol A	94.50%	192
Chitosan	TiO_2_/Chitosan-biochar composites	Co-calcination technique	600 °C/3 h	Rhodamine B	100%	193
Corn straw	ZnIn_2_S_4_/NiFe_2_O_4_/biochar composite	Pyrolysis	450 °C/2 h	Doxycycline hydrochloride	94.0%	194
Pine needle leaves	Cu–Fe bimetallic oxide/biochar/Ag_3_PO_4_ heterojunction	Pyrolysis/hydrothermal	750 °C/2 h	Phenol	20 mg per L phenol in 18 min	195
Pinewood powder	Biochar integrated Ag_3_PO_4_/α-Fe_2_O_3_ heterojunction	Pyrolysis/hydrothermal	900 °C/2 h	Tetracycline and ciprofloxacin	58.3% tetracycline and 79.4% ciprofloxacin in 2 h	196
Roots, stems and leaves of spartina alterniflora	Pure biochar	Pyrolysis	300–700 °C/2 h	Methylene blue	92.03%	197
Coconut shell fiber	Biochar decorated α-MnO_2_	Pyrolysis/hydrothermal	800 °C/3 h	Bisphenol A	100% in 90 min	198
Poplar woodchips	Mn@N-biochar	Impregnation-calcination method	500 °C/3 h	Sulfanilamide	98.3%	199
Corn straws	TiO_2_/biochar	Pyrolysis/hydrothermal	400–700 °C/2 h	Methyl orange	97.98%	200
Green sichuan pepper shells	Au/TiO_2_/biochar	Pyrolysis/hydrothermal	500 °C/2 h	Tetracycline	93.6%	201
Grapefruit peel powder	biochar/CdS–Fe_3_O_4_ nanocomposites	Pyrolysis/hydrothermal	180 °C/24 h	Chlorpyrifos	97%	202
Chitosan	WO_3_@TiO_2_/CS-biochar S-scheme heterojunction	Solid-phase co-calcination method	160 °C/24 h	Methylene blue and tetracycline	100% in 2 h	203
Corn straw	S–NaTaO_3_/biochar	Hydrothermal	200 °C/24 h	RhB, MO, acid orange 7, TCH, and CIP	RB (99.6%), MO (99.2%), acid orange 7 (84.5%), TCH (67.1%) and CIP (70.7%)	204
Murraya koenigii stem	Magnetic biochar-CeO_2_ nanocomposite	Pyrolysis/microwave-assisted precipitation	800 °C/2 h	Methyl orange	96.7%	205
Pine powder	Biochar/Co–Mo_2_C spheres/g-C_3_N_4_ nanocomposite	Pyrolysis	—	Tetracycline	98%	206
Coffee waste	α-NiMoO_4_/ZnFe_2_O_4_/biochar nanocomposite	Hydrothermal	—	Ketoprofen	98.65% in 3 h	207
Potato straw	BiWO_6_/TiO_2_/pyrochar and BiWO_6_/TiO_2_/hydrochar	Pyrolysis	500 °C/6 h	Sulfathiazole	—	208
Brewed waste coffee powder	Biochar/ZnO	Pyrolysis	200–800 °C/2 h	5-Methylbenzotriazole, carbamazepine, bisphenol A and ibuprofen	5-Methylbenzotriazole (>99%, 30 min), carbamazepine (70%, 60 min), bisphenol A (70%, 60 min) and ibuprofen (95%, 60 min)	209
Cattle dung	N-doped biochar/ZnO and Cu_2_O-based nanocomposites	Pyrolysis	700 °C/2 h	Doxycycline hydrochloride Cr(vi)	88.9% Cr(vi) and 92.9% doxycycline hydrochloride in 90 min	210
Pistachio shells	Biochar/CoFe_2_O_4_/Mn–Fe–LDH composite	Pyrolysis/co-precipitation	500 °C/2 h	Methylene blue and methyl orange	99.10% methylene blue and 99.89% methyl orange	211

#### 4.3.2 Photocatalytic removal of antibiotics

**Fig. 10:**
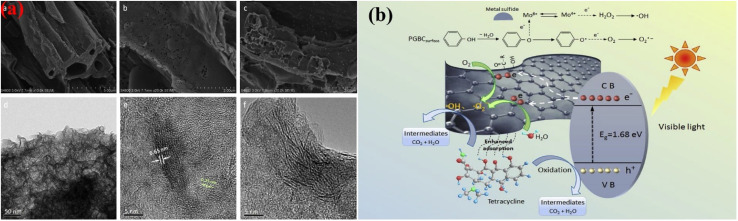
SEM pictures of biochar (a) [(a and b) and the g-MoS_2_ grafted biochar (c), TEM pictures (d), and HRTEM of g-MoS_2_ grafted biochar (e and f)]. Schematic of the degradation mechanism for TCA (b). Figure adapted/reproduced from ref. 212 with permission from Elsevier [(license number: 6145931330691) copyright 2025].

The green disposal of agricultural waste is realized by re-engaging the waste biomasses for useful applications based on the perception of “waste to wealth”. Straw-based biomass with polar hydroxyl and carbonyl functionalities is composed mainly of lignin, cellulose, and hemicellulose. The material is extremely beneficial for the construction of efficient photocatalysts, as it effectively improves charge transport and separation during photocatalysis. Ye *et al.* fabricated highly graphitized microtubular porous biochar from straw-based biomass.^212^ The fabricated biochar was grafted with g-MoS_2_ nanosheets to form a highly porous and graphitized photocatalyst for the eradication of tetracycline antibiotic (TCA). The SEM and TEM morphologies described the fabricated photocatalyst as a hollow and smooth microtubular structure with rich pores for the decoration and excellent dispersion of g-MoS_2_ with minimized agglomeration due to the chemical interaction between Mo and the functional groups of biochar, as shown in Fig. 10a. The inherited elongated porous network of straw-based biomass presents an excellent route for particle uptake and offers channels for charge conduction and transportation. The light absorption study of the samples indicated an increased absorption edge of visible light due to the possible C–S–Mo interaction in the nanocomposite. The charge separation and recombination techniques confirmed that the fabricated photocatalyst presented excellent charge separation for extended photocatalysis. It is accepted that the oxidation of toxic substances mainly depends on the mass transfer of the target materials and the light harvesting capability of the photocatalyst. The interaction of TCA with the surface of the photocatalyst at different pH values demonstrated that the maximum amount of TCA was adsorbed at a pH < 3 due to the electrostatic attraction between TCA and the fabricated photocatalyst. The trapping experiments confirmed that the generated oxygen species and holes were involved in the decomposition of the TCA under solar light irradiation. The introduction of highly graphitized biochar as a charge mediator provided a short diffusion length for the quick transportation and separation of the photogenerated charges to oxidize the target pollutant into simple molecules, as shown in Fig. 10b.

**Fig. 11:**
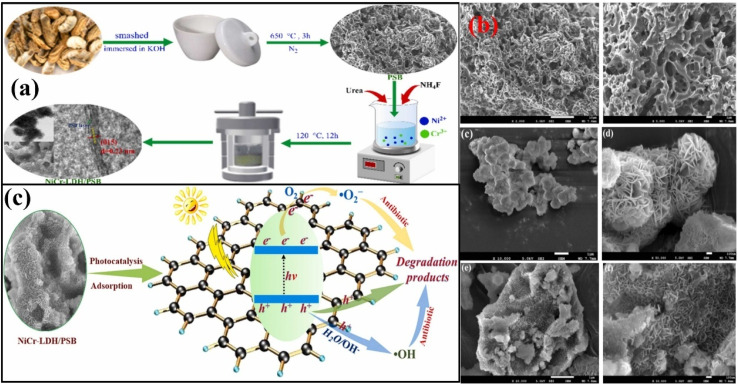
Schematic of the NiCrLHs/BC composites synthesis (a), SEM of pristine biochar (b) [(a and b), NiCrLHs (c and d) and NiCrLHs/BC (e and f)], and photocatalytic degradation mechanism of antibiotic over NiCr-LDH/PSB (c). Figure adapted/reproduced from ref. 213 with permission from Elsevier [(license number: 6145940732602) copyright 2025].

Both ofloxacin (OFN) and tetracycline antibiotics (TCA) were photocatalytically degraded by Feng *et al.* over the surface of NiCrLHs coupled biochar-based photocatalyst.^213^ Peanut shell-based biochar was loaded with NiCl_2_ and CrCl_3_ salts to form NiCrLHs/BC photocatalyst, as shown in Fig. 11a. The pristine biochar demonstrated hollow and porous morphology with enough fastening sites for the attachment of highly dispersed flower-like NiCrLHs to form lamellar NiCrLHs/BC nanocomposite, as shown in Fig. 11b. The photocatalytic degradations of both OFN and TCA were monitored under UV light irradiation. Interestingly, the rates of photocatalytic degradation were significantly improved for both antibiotics when the amount of biochar was increased in the nanocomposite, highlighting the significant role of biochar as a supportive material during photocatalysis. In the composite, biochar significantly reduced the agglomeration of NiCrLHs and improved charge conductivity, carrier separation and light sensitivity, advancing photocatalytic degradation. The degradation activities of both OFN and TCA were significantly reduced in the presence of alcohol and benzoquinone, which revealed the roles of hydroxyl and super oxide free radicals. The oxidation mechanism was explained according to the data generated, as shown in Fig. 11c. When irradiated under UV light, both biochar and NiCrLHs were excited to generate excited charges. The excited electrons were transferred immediately from NiCrLHs to biochar to improve charge separation and mobility for extended performance. The superior photocatalytic decomposition of both antibiotics was mainly attributed to the nanocomposite formation between biochar and NiCrLHs and enhanced charge separation due to the electron accepting nature of biochar. The biochar effectively validated the chemical reaction between the excited electrons and adsorbed oxygen to form super oxide free radicals for the degradation of both antibiotics. Table 3 illustrates a comparison of the adsorption and photocatalytic removal of pollutants with biochar-based materials.

**Table 3:** Comparison of adsorption and photocatalytic removal of pollutants with biochar-based materials

Photocatalyst/adsorbent	Method of preparation	Target pollutant	Adsorptive removal	Photocatalytic removal	Ref.
Iodine-doped biochar	Iodine doping technique	Phenol	33.60 mg g^−1^	92.9%	214
CuInS_2_ and MgO modified S-doped biochar	One-step pyrolysis	Rhodamine B	799.18 mg g^−1^	97.67%	215
La_2_S_3_ and MgO (La/Mg-biochar)	One-pot high-temperature sulfidation	Tetracycline hydrochloride	120.59 mg g^−1^	95.01%	216
S,N-codoped La_2_S_3_ modified biochar	One-pot sulfurization approach	Tetracycline hydrochloride	419.18 mg g^−1^	98.45%	217
Lignin-biochar (LC)/ZnAl_2_O_4_/BiPO_4_	Hydrothermal process	MB	74.1%	69.2%	218
GO/biochar/TiO_2_	Combination of biomass pyrolysis and the sol–gel method	Antibiotics	1.289 mg g^−1^	>95%	219
FeS/MgO-biochar	Pyrolysis	Rhodamine B	635.36 mg g^−1^	99.13%	220
biochar/NiCr-LDH composites	Hydrothermal method	Methyl orange	108.2 mg g^−1^	98.9%	221
CdS/Sulphur-modified biochar composites	Impregnation and pyrolysis method	Rhodamine B	864.46 mg g^−1^	99.18%	222
NiS–Mg/S-biochar	One-pot sulfurization/pyrolysis	Rhodamine B	802.49 mg g^−1^	96.98%	223
TiO_2_/biochar	Sol-hydrothermal method	Methyl orange	284.06 mg g^−1^	80.78%	224
N-doped biochar/PbMoO_4_	*In situ* precipitation	Tetracycline	20.10 mg g^−1^	88.25%	225
Cu_2_O nanoparticles/biochar	Precipitation/pyrolysis	Methyl orange	1610 mg g^−1^	94.5%	54
CuS–Mg/S-biochar	One-pot sulfurization process	Rhodamine B	981.67 mg g^−1^	95.70%	226
Fe_3_O_4_/CuWO_4_ loaded biochar	Precipitation/pyrolysis	Tetracycline	225.28 mg g^−1^	92.83%	227
Mn–CuO@biochar	Precipitation/pyrolysis	Congo red and eriochrome black T	5.26 and 4.17 mg g^−1^ respectively	98% and 95%	228
Fe_2_O_3_-loaded biochar	Precipitation/pyrolysis	Methylene blue	87.6%	70.8%	229
ZnFe_2_O_4_/biochar	One-pot microwave-assisted pyrolysis	Tetracycline hydrochloride	244.34 mg g^−1^	98.19%	230
TiO_2_-functionalized biochar composite	Pyrolysis/hydrothermal	Methyl orange	97.69%	99.47%	231
TiO_2_ supported biochar	Pyrolysis/precipitation	Ciprofloxacin	747.64 mg g^−1^	83.82%	232

#### 4.3.3 Photocatalytic removal of anions

**Fig. 12:**
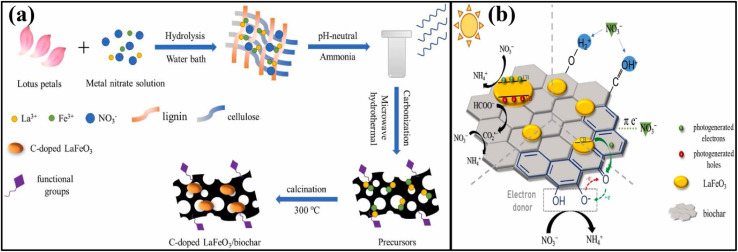
Schematic of the LFOBC fabrication (a). Schematic of the conversion of nitrate over LFOBC (b). Figure adapted/reproduced from ref. 233 with permission from Elsevier [(license number: 6145941017939) copyright 2025].

Biochar-based photocatalysts are not only used for the decomposition of organic dyes, antibiotics and metals but are also applied to eradicate various anions present in aqueous media. For instance, Liu *et al.* used biochar-based perovskite for the elimination and conversion of nitrate ions into useful ammonia product.^233^ The dried lotus-based biomass was pulverized and mixed with the nitrate salts of Fe and La to fabricate LaFeO_3_ incorporated biochar (LFOBC). The pH of the mixture was regulated at 7 with NH_4_OH and heated at 180 °C for 1.5 h. The product obtained was heated at 300 °C to give the final LFOBC photocatalyst, as shown in Fig. 12a. The fabricated photocatalysts showed a highly porous morphology due to the liberation of gases during microwave-based pyrolysis. The pore formation was assisted by the hydrolysis of cellulose and hemicellulose of biochar using La^3+^ and Fe^+^ ions, as confirmed by the FTIR peaks of the –OH groups. All particles of LaFeO_3_ strongly adhered to the surface and in the pores of biochar. The XPS study of the composite demonstrated a relatively high ratio of carbon–oxygen bonds to show that the surface of the biochar influenced abundant oxygen carrying groups. A lower ratio of *I*_D_/*I*_G_ from the Raman spectra confirmed that the composite was more graphitized and aromatized by the hydrolysis of metal ions. Light absorption of the composite was higher than that of the pristine biochar and LaFeO_3_, indicating that the arrival of biochar assisted light absorption for the production of workable charges. When applied for the conversion of nitrate to NH_4_^+^, the nanocomposite showed enhanced removal efficiency compared to pristine biochar and LaFeO_3_. Additionally, the rate of NH_4_^+^ was highly accelerated when the amount of biochar increased. These enhanced activities were attributed to the enhanced charge separation, large surface area and prolonged light absorption by the nanocomposite. A high selectivity for NH_4_^+^ ions was attributed to the presence of a phenolic functional group on the surface of the biochar. A schematic, as shown in Fig. 12b, was provided to show the removal of nitrate ions through the selective formation of NH_4_^+^ ions. The accumulation and adsorption of nitrate ions on the rich porous surface of the nanocomposite were controlled by different interactions between the nitrate ions and the nanocomposite. The photocatalytically excited electrons were immediately conveyed to the surface due to the high graphitization of biochar to reduce nitrate ions to NH_4_^+^ with the aid of a proton. The FTIR spectra of the nanocomposite before and after adsorption of the nitrate ions were conducted using HNO_3_ as the adsorbate to investigate the mechanism behind nitrate conversion. Interestingly, the FTIR spectra of the nanocomposite before and after the adsorption of the nitrate ions were highly analogous. The peaks related to C=O, O–H and C–OH were markedly weakened, suggesting that the reductive functional groups of biochar were moderately oxidized, which worked as electron donors to reduce nitrate into NH_4_^+^. A remarkable weakening of the aromatic stretching peaks suggested that π-electrons were firmly attached to the nitrate ions. Again, enhanced redox electrochemical curves were found in the case of the nanocomposite, demonstrating accelerated reduction of nitrate to NH_4_^+^. The XPS study of the nanocomposite before and after adsorption of the nitrate ions suggests that the proportion of C–OH was reduced, while that of O–C=O increased, indicating that the reductive groups of biochar provided electrons for the reduction of nitrate ions into NH_4_^+^.

## 5. Relationship between adsorptive and photocatalytic removal of pollutants

**Fig. 13:**
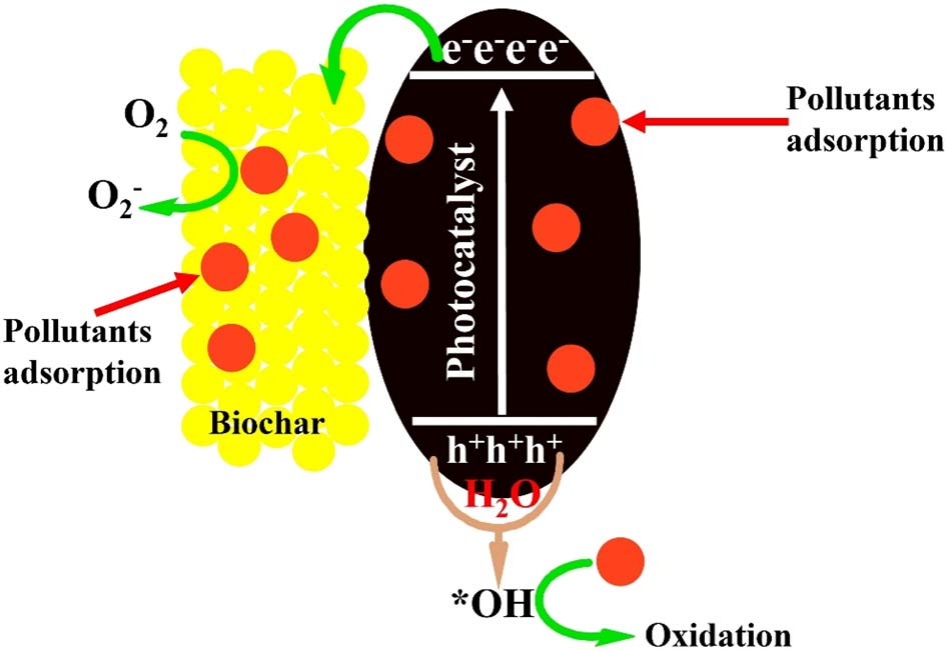
Schematic showing the relationship between adsorption and photocatalytic processes.

The process of photocatalysis is significantly dependent on the adsorption process. The photocatalytic degradation of pollutants or the reduction of metals, and the reduction of anions are highly dependent on the adsorption of target pollutants. It is accepted that the oxidation/reduction of pollutants depends on the generation of excited charges and degrading species, such as super oxide anions and hydroxyl free radicals. The generation of these degrading species depends on the adsorption of molecular oxygen and water. If molecular oxygen and water are properly adsorbed on the surface of the photocatalysts, the rate of generation of the degrading species is large and hence the removal of the pollutants is fast^83^^.^ If the pollutants are directly oxidized by VB holes, then their prior adsorption is required before their degradation. It has been found that if photocatalysts have a large specific surface area for the adsorption of target materials, their photocatalytic efficiencies for eradicating pollutants are quite impressive. Thus, it is concluded that whether the eradication of pollutants is directly manifested by the holes produced in the photocatalyst with the courtesy of absorbed solar light, their high adsorption is related to enhanced degradation efficiency. If pollutants are indirectly degraded by the produced super oxide anions and hydroxyl free radicals, the proper adsorption of molecular oxygen and water guarantees enhanced pollutant eradication efficiency.^234^ A schematic showing the relationship between adsorption and photocatalytic processes is depicted in Fig. 13.

## 6. Conclusion, perspective and outlook

In this review article, we discuss the role of biochar in the purification of the environment. Biochar is prepared from waste biomasses. It is a major porous material that can be used to adsorb toxic materials from wastewater and soil effectively. It is also used to purify and remove toxins from the environment through the courtesy of solar light-driven photocatalysis. Here, we discuss the characteristics of biochar and its role in photocatalysis. The adsorptive and photocatalytic removal of organic and inorganic pollutants, antibiotics, heavy metals, nuclear materials, nitrates and phosphates using biochar-based adsorbents and photocatalysts are discussed in detail. The mechanisms behind the adsorptive and photocatalytic removal of pollutants governed by biochar are elaborated to understand the chemistry of pollutant removal under different conditions.

The applications of biochar have been significantly highlighted by many authors in recent decades. It has been derived from both plant and animal-based biowastes. However, investigation related to biochar performance is still limited to the laboratory, and its real-life out-door field applications and field trials are limited. Therefore, further studies should focus on demonstrating its application on a large scale. The decay of biomasses in crop fields significantly alters the pH of the soil, which blocks the availability of many nutrients to plants. Therefore, alternative methods should be chalked out to address this issue. The long-term applications and stability of biochar-based adsorbents and photocatalysts are still under question. Numerous studies have demonstrated that the adsorption and photocatalytic efficiencies of biochar modified with foreign substances are reduced after successive cycles due to the blockage of pores, significant reduction in active sites and leaching of oxygen and nitrogen functional groups. Therefore, the stability of biochar in terms of adsorption/desorption, photocatalytic degradation and bio-accessibility must be highlighted in future studies. Further, the cost of modified biochar-based materials for the adsorptive and photocatalytic removal of pollutants poses a huge barrier to their practical applications. Currently, biochar is generally prepared under high temperature conditions; therefore, its synthesis procedure must be adjusted to subside risks, such as airborne dispersal of small biochar particles causing health issues, particularly chest problems in infants. Another issue related to the use of biochar is the alteration in the habitat of microbial species. Therefore, studies must focus on investigating the effect of biochar on the diversity and activity of microbial species in a biochar-based environment. This requires an inclusive ecotoxicological investigation linking soil organisms to simplify ecological risk calculations. The adhesive and photocatalytic performances of biochar-based materials are primarily related to their surface area, pore volume, and the presence of certain functional groups. A large surface area with a huge pore volume generally presents numerous active sites for the adsorption and photocatalysis of toxins. Again, several factors, including temperature and experimental pH, largely alter the performance of biochar-based materials. The presence of certain functionalities controls the adsorption and photocatalysis of toxins; therefore, more efforts should be devoted to investigating the pollutant removal mechanism with biochar-based materials. The employment of biochar-based adsorbents and photocatalysts significantly alters the total dissolved organic carbons and salts and the total suspended solids of water. Therefore, after applying biochar-based materials, these factors must be monitored thoroughly through repetitive remote sensing procedures, on-site determination, and computer-based water quality checking methods. Machine learning in the proper design of biochar is an excellent tool for promoting biochar-based technology. Sharing machine-generated algorithmic models or hybrid models within the community will extend the significance of biochar-based adsorbents/photocatalysts. For this, the development of machine-generated models with experimental justification will serve as inverse guidance for the wide application and synthesis of biochar-based adsorbents/photocatalysts. A hybrid machine learning model based on the optimization framework should be recognized to fill the gap between the preparation of biochar-based adsorbents/photocatalysts and their performance. This hybrid framework will provide excellent feedstock and the best preparation conditions to remove the target pollutants. In the future, multi-scaled structural investigation by employing physical and chemical modifiers, introducing metals and non-metal dopants and functionalizing biochar-based materials is highly suggested to enhance the adsorption and photocatalytic degradation of hazardous materials present in different segments of our environment.

## Conflicts of interest

There is no conflict of interest.

## Data Availability

This article is a review and does not report any new data.
